# The Gastrointestinal Barrier—Mechanisms of Barrier Dysfunction in Liver Cirrhosis and Spontaneous Bacterial Peritonitis

**DOI:** 10.3390/biomedicines14051084

**Published:** 2026-05-11

**Authors:** Catalina Olaru-Stavila, Sara Martina Steinmann, Patricia Mester, Martina Müller, Eugen Tcaciuc, Karsten Gülow

**Affiliations:** 1 Department of Gastroenterology, Nicolae Testemitanu State University of Medicine and Pharmacy, MD-2004 Chisinau, Moldova; colaru38@gmail.com; 2 Department of Internal Medicine I, Gastroenterology, Hepatology, Endocrinology, Rheumatology, Immunology, and Infectious Diseases, University Hospital Regensburg, 93053 Regensburg, Germany; sara.steinmann@ukr.de (S.M.S.); patricia.mester-pavel@klinik.uni-regensburg.de (P.M.); martina.mueller-schilling@ukr.de (M.M.)

**Keywords:** liver cirrhosis, gastrointestinal barrier, bacterial translocation, spontaneous bacterial peritonitis (SBP), gut–liver axis, cirrhosis-associated immune dysfunction (CAID), inflammation, epithelium

## Abstract

The gastrointestinal (GI) barrier is a highly coordinated, multilayered defence system that maintains intestinal homeostasis by separating the luminal microbiota from the internal milieu. In liver cirrhosis, this barrier undergoes profound structural and functional disruption, emerging as a central driver of bacterial translocation and infection-related complications. Among these, spontaneous bacterial peritonitis (SBP) represents a major determinant of morbidity, mortality, and disease progression. Barrier failure in cirrhosis is not attributable to a single defect but results from the convergence of multiple interconnected mechanisms. Structural alterations include disruption of epithelial tight junctions and deterioration of the mucus layer, leading to increased intestinal permeability and loss of spatial compartmentalisation. These changes are compounded by microbial dysbiosis, characterised by reduced diversity, depletion of short-chain fatty acid-producing taxa, and expansion of pathobionts. In parallel, cirrhosis-associated immune dysfunction impairs both mucosal and systemic antimicrobial defences, while gut–vascular barrier disruption facilitates systemic dissemination of bacteria and microbial products. The resulting increase in bacterial translocation plays a pivotal role in the pathogenesis of SBP and contributes to systemic inflammation, circulatory dysfunction, and acute decompensation. Importantly, this process establishes a self-amplifying pathogenic loop in which barrier dysfunction, dysbiosis, and immune dysregulation mutually reinforce each other. Recent advances have identified key molecular pathways involved in barrier regulation, including bile acid–FXR signalling and microbiome-derived metabolites, providing novel targets for therapeutic intervention. While current management relies largely on antibiotics and supportive care, emerging strategies aim to restore barrier integrity and modulate the gut–liver axis. A deeper understanding of GI barrier dysfunction offers new opportunities to prevent bacterial translocation and improve clinical outcomes in patients with liver cirrhosis.

## 1. Introduction

Liver cirrhosis represents the common end stage of chronic liver diseases and is characterised not only by progressive architectural distortion and portal hypertension but also by progressive systemic immune alterations. Among the most clinically relevant complications of cirrhosis are bacterial infections, particularly spontaneous bacterial peritonitis (SBP), which significantly increases short-term mortality and frequently precipitates acute-on-chronic liver failure. A central driver of these infectious complications is dysfunction of the gastrointestinal (GI) barrier [1,2,3,4,5,6,7].

The GI barrier is a highly organised, multilayered defence system that separates the luminal microbiota from the sterile internal milieu. It consists of the epithelial layer, including its tight junction complexes, the overlying mucus layer, antimicrobial peptides, secretory immunoglobulin A (sIgA), and a finely regulated mucosal immune system. Under physiological conditions, this integrated barrier allows nutrient absorption while preventing microbial translocation and uncontrolled immune activation.

In liver cirrhosis, this equilibrium becomes progressively disrupted. Portal hypertension induces structural and hemodynamic alterations in the intestinal wall, including congestion, edema, and impaired microcirculation. At the same time, cirrhosis is associated with profound immune dysregulation, characterised by simultaneous systemic inflammation and immune paresis. These changes impair epithelial tight junction integrity, reduce mucosal immune competence, and alter mucus composition. Concomitantly, dysbiosis of the intestinal microbiome further destabilises barrier function and favours the expansion of pathogenic bacterial species [2,4,8].

Loss of barrier integrity facilitates bacterial translocation from the intestinal lumen to mesenteric lymph nodes and, ultimately, into the systemic circulation and ascitic fluid. In the setting of cirrhosis with ascites, this process constitutes the key pathogenic mechanism underlying SBP. Importantly, bacterial translocation is not merely a consequence of advanced disease but actively contributes to systemic inflammation, circulatory dysfunction, and clinical decompensation [2,4,5,9,10,11].

A comprehensive understanding of GI barrier organisation and the mechanisms driving its failure in cirrhosis is therefore essential. Elucidating the interplay between epithelial integrity, immune homeostasis, portal hypertension, and microbiome alterations may not only clarify the pathogenesis of SBP but also uncover novel therapeutic strategies aimed at restoring barrier function and preventing infectious complications [2,9].

This review summarises the structural organisation of the GI barrier, delineates mechanisms of barrier dysfunction in liver cirrhosis, and discusses how these alterations contribute to bacterial translocation and spontaneous bacterial peritonitis. It also provides a brief overview of microbiome alterations and emerging therapeutic perspectives.

## 2. Organisation of the Gastrointestinal Barrier

**Figure 1:**
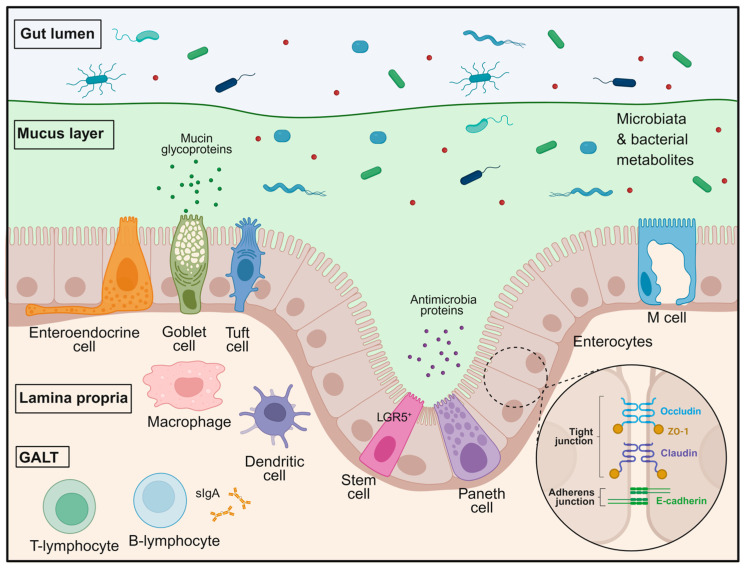
Intestinal barrier architecture. The intestinal barrier is composed of a mucus layer, a polarised epithelial monolayer, and the underlying lamina propria. The epithelium includes enterocytes, goblet cells, Paneth cells, enteroendocrine cells, and M cells, linked by tight junctions. The lamina propria harbours resident immune cells, including macrophages, dendritic cells, and lymphocytes, supporting immune surveillance. Inset: schematic representation of epithelial junctional complexes, including tight junctions (occludin, claudins, ZO-1) and adherens junctions (E-cadherin). GALT, gut-associated lymphoid tissue; LGR5^+^, leucine-rich repeat G-protein coupled receptor 5-positive; ZO-1, zonula occludens-1. The figure was created in BioRender. Pollinger, K. (2026) https://BioRender.com/lln5uwf.

The GI barrier represents a complex and highly coordinated defence system that separates the luminal environment from the internal milieu while permitting selective absorption of nutrients, water, and electrolytes. This barrier is organised into multiple functional layers that work in concert to maintain intestinal homeostasis and prevent microbial invasion. Structurally, it comprises the intestinal epithelium, the mucus layer, antimicrobial peptides, immune cells within the lamina propria, and the gut-associated lymphoid tissue [12,13,14,15,16,17]. More recent concepts additionally emphasise that the intestinal barrier should be understood as a dynamic and integrated system in which epithelial integrity, mucus organisation, microbial signals, and mucosal immune responses are functionally interconnected rather than acting as isolated defence layers (Figure 1) [14,18].

Under physiological conditions, the GI barrier maintains a tightly regulated equilibrium between tolerance to commensal microbiota and rapid defence against potentially pathogenic microorganisms. The intestinal epithelium forms the first physical line of defence, while secreted mucus and antimicrobial factors limit direct contact between microbes and epithelial cells and represent the chemical line of defence. Beneath the epithelial layer, innate and adaptive immune mechanisms provide an additional level of surveillance and response. Disruption of any of these components, often driven by dysbiosis and portal hypertension, can compromise barrier integrity and facilitate microbial translocation, a process that becomes particularly relevant in pathological conditions such as liver cirrhosis [1,2,4,19]. This concept has gained increasing relevance in recent literature, which describes the intestinal barrier as a highly regulated interface controlling not only microbial containment but also inflammatory tone, epithelial restitution, and host–microbiome crosstalk [14,18].

### 2.1. Epithelial Integrity and Tight Junctions

The intestinal epithelium forms a single layer of polarised epithelial cells that separates the luminal environment from the internal milieu. Despite its minimal thickness, this epithelial lining constitutes a highly effective physical barrier that simultaneously permits selective nutrient absorption while restricting the passage of microorganisms and luminal toxins [12,14,18,20,21,22].

The intestinal epithelium is a vital semipermeable interface, organised into crypt units, with protrusions called villi found specifically in the small intestine to maximise nutrient absorption. It comprises diverse cell types, including enterocytes, mucin-producing goblet cells, enteroendocrine cells, Paneth cells, stem cells, tuft cells, and M cells (Figure 1). This architectural arrangement minimises the exposure of the proliferative crypt base to luminal contents, where long-lived LGR5^+^ stem cells drive a rapid cellular turnover of approximately three to seven days, a process essential for ensuring barrier integrity and constant renewal of the epithelial surface [14,18,23]. Complementing this structural framework, specialised cell types such as goblet cells and Paneth cells, situated at the base of the intestinal crypts, provide a physicochemical shield by secreting mucus and antimicrobial peptides, thereby preserving the spatial segregation of luminal microbes from the host tissue [14,18]. Recent work further highlights that epithelial barrier maintenance depends not only on cell replacement and junctional sealing, but also on coordinated epithelial repair programmes that link proliferation, survival, and restitution to tight-junction-associated signalling [18,20].

Barrier integrity is largely maintained by intercellular junctional complexes that connect adjacent epithelial cells. These complexes include tight junctions, adherens junctions, and desmosomes, which collectively provide structural cohesion and regulate paracellular permeability. Among these structures, tight junctions represent the most apical component of the junctional complex and are central to the regulation of epithelial barrier function (Figure 1) [1,2,4,19,24].

Molecularly, tight junctions consist of a network of transmembrane proteins, including claudins, occludins, and junctional adhesion molecules, which are anchored to cytoplasmic scaffold proteins such as the zonula occludens (ZO) family. In turn, ZO proteins bind the junctional complex to the perijunctional actomyosin ring. Through interactions with the actin cytoskeleton, these complexes dynamically regulate the permeability of the paracellular pathway [20,25,26]. Importantly, tight-junction proteins are now recognised not merely as static sealing elements but as multifunctional regulators of epithelial homeostasis, contributing to cell survival, wound healing, and barrier restitution in addition to paracellular permeability control [20,25].

Tight junctions must adapt to the varying demands of the intestine, both maintaining a seal and permitting paracellular transport. Paracellular flux across tight junctions occurs via two principal routes: the high-capacity, charge-selective pore pathway and the lower-capacity non-selective leak pathway [25,27]. This functional distinction between pore and leak pathways has been reaffirmed in recent reviews and remains highly relevant for disease states, because pathological barrier dysfunction is often driven less by complete junctional disruption than by selective dysregulation of these permeability routes [25]. Pore pathway permeability is primarily determined by claudin proteins, the key structural components of tight junctions. Despite their structural similarities, claudins can be functionally divided into channel-forming and barrier-forming types. Channels formed by claudin-2 and claudin-15 are both size-selective and cation-selective, facilitating the paracellular transport of cations and water. In contrast, tightening claudins such as claudin-1 and claudin-3 are more abundant in the distal intestine, where high microbial loads require a more robust barrier function. The tight junction leak pathway allows passage of larger, non-selective solutes across the epithelial barrier and, when compromised, is associated with large-scale movement of luminal contents, including bacterial products [21,26,28]. Activation of the leak pathway is a regulated process driven by cytoskeletal tension. Circumferential contraction of the perijunctional actomyosin ring is controlled by phosphorylation of the myosin regulatory light chain modulated by myosin light chain kinase. This contraction pulls on anchored tight junction proteins, widening paracellular pathways and thereby increasing permeability. Tight junctions are therefore not static structures but respond to a variety of physiological and pathological stimuli, including inflammatory cytokines, microbial products, and metabolic signals [14,25,29,30]. Accordingly, current models describe tight-junction regulation as a highly dynamic process integrating cytoskeletal remodelling, junctional protein trafficking, and context-dependent epithelial signalling [20,25].

Disruption of tight junction organisation leads to increased intestinal permeability, facilitating the passage of bacteria and microbial products across the epithelial barrier. In the context of liver cirrhosis, several factors, including chronic inflammation, oxidative stress, portal hypertension, and alterations of the intestinal microbiota, have been implicated in the impairment of epithelial junctional integrity. These changes contribute to enhanced bacterial translocation, a process that plays a key role in the development of infectious complications such as spontaneous bacterial peritonitis [1,2,4,9,31,32,33]. Given this broader understanding, epithelial tight-junction failure in cirrhosis should be viewed not only as a structural defect, but also as a disturbance of epithelial resilience and repair capacity, which may further amplify susceptibility to bacterial translocation [20,25].

### 2.2. The Mucus Layer as a Protective Interface

In addition to epithelial junctional complexes, the mucus layer represents a key structural and functional component of the GI barrier (Figure 1). This viscoelastic gel covers the epithelial surface throughout the gastrointestinal tract and functions as the first point of contact between the host tissue and the luminal environment. By creating a physical separation between microorganisms and epithelial cells, the mucus layer plays a central role in limiting microbial access to the epithelium and maintaining intestinal homeostasis [13,16,34]. Recent literature further emphasises that the mucus layer is not merely a passive physical coating but an active regulatory interface that shapes microbial spatial organisation, concentrates host-defence molecules, and contributes directly to mucosal immune homeostasis [35,36].

The mucus layer is mainly composed of large, heavily glycosylated mucin proteins that are secreted by specialised epithelial goblet cells. Among these, the gel-forming mucin MUC2 represents the predominant structural component of intestinal mucus. Following secretion, mucins rapidly expand and assemble into a complex three-dimensional polymeric network that confers viscosity and elasticity to the mucus layer. This matrix traps microorganisms, microbial products, and particulate matter while allowing diffusion of nutrients and small molecules toward the epithelial surface [16,34]. More recent work has also highlighted that goblet cells actively coordinate barrier defence through regulated mucus release and interaction with immune pathways, thereby linking epithelial stress responses to mucus renewal and antimicrobial protection [35,37].

In the small intestine, mucins form a loose mucus layer, whereas in the colon, the mucus barrier is organised into two distinct layers. The inner mucus layer is dense and firmly attached to the epithelium and is normally sterile, devoid of bacteria, thereby forming an effective barrier that prevents microbial contact with epithelial cells. In contrast, the colonised outer mucus layer is less compact and provides a habitat for commensal microorganisms. This spatial organisation allows controlled coexistence between the host and the intestinal microbiota while limiting direct epithelial exposure to microbial stimuli [14,38,39,40].

Beyond its mechanical function, the mucus layer also represents an active biochemical defence system. It contains a variety of host-derived molecules, including antimicrobial peptides such as C-type lectin RegIIIβ, defensins, lysozyme, and sIgA, which help regulate microbial growth and composition. Secretory IgA primarily mediates immune exclusion. It is transcytosed across the epithelium and trapped in mucus, creating an antigenic barrier that prevents pathogen colonisation. Moreover, the mucus matrix provides a scaffold that concentrates antimicrobial and immune mediators near the epithelial surface, maintaining spatial segregation and thereby reinforcing local immune defence and contributing to mucosal immune homeostasis (Figure 1) [22,41,42].

Maintenance of the gastrointestinal mucosal barrier depends on bile acid signalling. Primary bile acids activate the farnesoid X receptor (FXR) to upregulate the expression of the gel-forming mucin MUC2. This signalling axis, often supported by the membrane receptor TGR5, reinforces the biochemical and mechanical integrity of the mucus matrix [5,19,43]. In liver cirrhosis, altered bile acid metabolism and impaired FXR signalling have been implicated as central contributors to mucus barrier disruption. Importantly, more recent human data indicate that intestinal FXR–FGF19 signalling is dysregulated in patients with cirrhosis and correlates with impaired intestinal defence, supporting the clinical relevance of defective bile acid signalling in advanced liver disease [43,44]. Direct evidence that FXR loss reduces MUC2 expression, however, derives primarily from experimental models [5].

In liver cirrhosis, the mucus barrier undergoes substantial structural and functional alterations. Experimental and clinical studies indicate that the mucus layer becomes significantly thinner and structurally impaired, accompanied by reduced goblet cell density and decreased expression of the major mucin MUC2. Notably, these changes appear to be driven primarily by cirrhotic pathophysiology rather than by portal hypertension-induced congestion alone, as experimental models of pre-hepatic portal hypertension maintain an intact mucus barrier, whereas cirrhotic animals exhibit pronounced mucus layer deterioration [5,43,45].

The mechanisms underlying mucus barrier dysfunction in cirrhosis are multifactorial. Altered bile acid metabolism and impaired signalling through the FXR have been implicated as central contributors to mucus barrier disruption, which compromises the sterile status of the inner mucus layer [43,46]. Importantly, although experimental studies support a mechanistic link between impaired FXR signalling and reduced mucin expression, the evidence that loss of FXR activity directly contributes to reduced MUC2 expression derives primarily from animal models. In contrast, human data mainly indicate that intestinal FXR–Fibroblast Growth Factor 19 (FGF19) signalling is dysregulated in patients with cirrhosis and correlates with impaired intestinal defence, supporting the concept that defective bile acid signalling is clinically relevant in advanced liver disease, while direct evidence for FXR-dependent MUC2 downregulation in humans remains limited [5,44,47] (Zitat). At the same time, cirrhosis is associated with profound intestinal dysbiosis characterised by depletion of beneficial microbial metabolites, including short-chain fatty acids (SCFAs), which may further impair goblet-cell function and mucin production, thereby contributing to mucus layer thinning [48,49,50]. This interaction between bile acid dysregulation, microbial metabolite depletion, and goblet-cell dysfunction is increasingly viewed as a key mechanism linking dysbiosis to loss of mucus integrity in cirrhosis [43].

Deterioration of the mucus barrier has direct consequences for host–microbe interactions. When the inner mucus layer becomes thinner and loses its normally bacteria-free status, microorganisms can penetrate deeper into the mucus matrix and establish close contact with the epithelial surface. This loss of spatial compartmentalisation promotes epithelial exposure to microbial products and facilitates pathological bacterial translocation across the intestinal epithelium into the portal circulation [8,51]. Defective mucus renewal and enhanced mucus degradation can aggravate barrier failure by facilitating direct host–microbe contact and persistent inflammatory activation [52,53].

Importantly, mucus barrier dysfunction does not occur in isolation but acts in concert with other barrier defects associated with cirrhosis. In addition to epithelial tight junction disruption, cirrhosis has been shown to impair the gut–vascular barrier, resulting in increased endothelial permeability within the intestinal microvasculature. The gut–vascular barrier serves as a secondary line of defence, preserving structural compartmentalisation against microorganisms that have breached the intestinal epithelial layer. Impaired FXR signalling, dysbiosis, and altered bile acid metabolism disrupt the gut–vascular barrier, facilitating the systemic translocation of microbial products into the portal circulation, thereby driving systemic inflammatory responses and aggravating portal hypertension [5,10,31,51].

Together, these defects create multiple levels of barrier failure that collectively promote bacterial translocation. The degree of intestinal barrier impairment appears to worsen with advancing stages of cirrhosis and correlates with systemic endotoxemia and inflammation [1,8,51].

Clinically, these processes have profound implications. Bacterial translocation, most commonly involving *Enterobacteriaceae* or *Streptococcaceae* species, represents a key pathogenic mechanism underlying spontaneous bacterial peritonitis, bacteremia, and other infection-related complications of cirrhosis. Reduced microbial diversity has also been associated with an increased risk of hepatic encephalopathy and spontaneous bacterial peritonitis. Furthermore, systemic inflammation triggered by microbial translocation can aggravate portal hypertension by promoting intrahepatic microvascular dysfunction and activation of hepatic stellate cells, thereby reinforcing a pathogenic feedback loop between intestinal barrier dysfunction and progressive liver disease. This pathogenic loop is fundamental to the development of spontaneous bacterial peritonitis and hepatic encephalopathy [1,2,4,9].

From a therapeutic perspective, strategies aimed at restoring intestinal barrier integrity have gained increasing attention. Experimental studies suggest that pharmacological activation of FXR may improve mucus barrier function and reduce bacterial translocation via the portal circulation. In addition, microbiome-targeted approaches—including lactulose, rifaximin, probiotics, and faecal microbiota transplantation—have been explored as potential strategies to restore microbial balance and improve barrier function, although their clinical efficacy appears to vary depending on disease stage and microbiome composition [54,55,56,57]. Given the growing literature on bile acid signalling, future therapeutic concepts will likely need to distinguish more clearly between direct epithelial effects, mucus-restoring effects, and broader gut–liver immune-metabolic effects of FXR-targeted interventions [43,44].

## 3. Immune Homeostasis and Immune Dysregulation

Beyond serving as a physical barrier, the intestinal epithelium plays a central role in maintaining immune homeostasis by coordinating tolerance toward commensal microorganisms and food-derived antigens while enabling rapid responses to invading pathogens. This balance requires continuous communication between epithelial cells, the gut-associated lymphoid tissue (GALT), and the intestinal microbiota (Figure 1). Multiple components of the GI barrier contribute to this equilibrium, including the chemical barrier, comprising mucus, antimicrobial peptides, and secretory immunoglobulin A (sIgA), and the physical barrier formed by the epithelial monolayer and intercellular junctions. In a state of eubiosis, these layers act synergistically to prevent the translocation of viable bacteria and excessive exposure to microbe-associated molecular patterns (MAMPs), such as lipopolysaccharide (LPS), flagellin, and bacterial nucleic acids [14,18,22]. Current concepts extend this view by emphasising that intestinal immune homeostasis in cirrhosis is shaped not only by barrier disruption itself, but also by persistent microbial stimulation that progressively reprograms both mucosal and systemic immune responses [58,59].

Detection of microbial signals is mediated by pattern recognition receptors (PRRs), which induce both transcriptional and non-transcriptional responses. Intestinal epithelial cells express Toll-like receptors (TLRs) and nucleotide-binding oligomerisation domain (NOD)-like receptors, as well as cytosolic DNA and RNA sensors, including cGAS and RIG-I-like helicases. Importantly, immune tolerance is not determined solely by ligand recognition but also by the compartmentalisation of these receptors. In healthy conditions, key receptors such as TLR4 and TLR5 are predominantly localised to the basolateral membrane or intracellular compartments, ensuring that luminal commensals are largely ignored while invasive microbes are rapidly detected [6,14]. In cirrhosis, however, chronic exposure to translocating microbial products is thought to disturb this finely regulated sensing architecture, thereby contributing to maladaptive immune activation on the one hand and immune hyporesponsiveness on the other [58,59].

TLR signalling pathways are central to epithelial barrier function. Activation of TLRs induces NF-κB-dependent transcriptional programmes via adaptor proteins such as MYD88 and TRIF, regulating tight junction integrity, mucus secretion, antimicrobial peptide production, and epithelial repair mechanisms. In cirrhosis, however, TLR signalling appears to be dysregulated in a receptor-, cell-type-, and context-dependent manner rather than uniformly increased or decreased. TLR4 has been most consistently linked to persistent exposure to gut-derived LPS and maladaptive inflammatory signalling, whereas data on TLR2 and TLR5 are more heterogeneous and likely depend on the intestinal compartment and disease stage. At the same time, chronic microbial stimulation may impair effective downstream signalling responses, including MYD88-dependent antimicrobial defence, thereby contributing to reduced goblet-cell function, diminished mucus production, and downregulation of tight junction proteins such as occludin and ZO-1 (Zitat) [5,7,60,61,62]. This dysregulation is increasingly interpreted as part of a broader immune-reprogramming process in advanced liver disease, in which sustained innate immune triggering coexists with impaired effector function [58,59,63].

In parallel, cytosolic sensing pathways mediated by NOD1 and NOD2 are essential for maintaining epithelial homeostasis. NOD2, in particular, regulates Paneth cell function and defensin secretion, which are critical for maintaining crypt sterility and controlling bacterial overgrowth. Impairment of NOD signalling, as observed in cirrhosis or in individuals carrying NOD2 loss-of-function variants, disrupts this protective mechanism and facilitates bacterial access to the epithelial surface and underlying vasculature [64,65].

Secretory IgA further contributes to mucosal immune defence by mediating immune exclusion. By coating microorganisms and antigens, sIgA prevents microbial adherence to epithelial cells and limits penetration into the mucosa. In cirrhosis, disruption of this mechanism—potentially exacerbated by microbiota capable of degrading immunoglobulins—facilitates closer microbial–epithelial interactions and promotes bacterial translocation [42,66].

In addition to microbial sensing, the intestinal epithelium functions as a metabolic sensor through G-protein-coupled receptors that detect microbial metabolites such as short-chain fatty acids (SCFAs). SCFAs, particularly butyrate, support epithelial energy metabolism, enhance tight junction integrity, and promote regulatory T-cell differentiation. In cirrhosis, dysbiosis leads to depletion of SCFA-producing bacteria, resulting in metabolic impairment of epithelial cells, reduced IL-10 production, and further loss of barrier integrity [48,50,67].

Immune regulation within the GALT is orchestrated by dendritic cells, macrophages, and lymphocyte populations. Under physiological conditions, dendritic cells exhibit a tolerogenic phenotype, promoting Treg differentiation via retinoic acid and TGF-β signalling. In cirrhosis, however, dendritic cell function shifts toward a pro-inflammatory phenotype, with increased recruitment of Th1 and Th17 cells. At the same time, impaired migration of dendritic cells due to downregulation of CCR7 and structural alterations of lymphoid tissues compromises effective antigen presentation and contributes to immunoparesis [6,7]. Accordingly, mucosal immune dysfunction in cirrhosis should not be understood as a simple loss of defence, but rather as a state of ineffective, spatially dysregulated immune activation with impaired antimicrobial effector function [58,59,68].

These local immune alterations are closely linked to systemic immune dysfunction. In cirrhosis, the immune system undergoes profound remodelling, resulting in cirrhosis-associated immune dysfunction (CAID), a paradoxical syndrome characterised by the coexistence of systemic inflammation and immune paralysis. Early stages are dominated by low-grade inflammation with preserved immune function, whereas advanced disease and acute-on-chronic liver failure are characterised by severe systemic inflammation combined with profound immune suppression and increased infection risk [2,6,7,59]. CAID is best understood as a dynamic and stage-dependent process rather than a fixed immune phenotype, characterised by a progressive transition from chronic immune activation to immune exhaustion, defective antimicrobial effector responses, and heightened susceptibility to infection and organ failure [6,59,69].

A central contributor to CAID is impaired hepatic immune surveillance. Kupffer cells, which normally clear gut-derived bacteria from the portal circulation, exhibit reduced phagocytic capacity and dysregulated cytokine responses in cirrhosis. Concurrently, decreased hepatic synthesis of complement proteins compromises opsonisation and bactericidal activity. These defects are particularly relevant in ascitic fluid, where reduced complement levels markedly impair antimicrobial defence [7,70,71].

Persistent bacterial translocation further drives immune dysregulation by exposing the immune system to PAMPs such as LPS and bacterial DNA. Chronic stimulation leads to sustained systemic inflammation but also induces immune exhaustion. Neutrophil dysfunction is a hallmark of this process, characterised by impaired chemotaxis, reduced phagocytosis, and diminished intracellular oxidative burst. Paradoxically, increased extracellular release of reactive oxygen species (ROS) may contribute to systemic oxidative stress and further damage epithelial and vascular barriers (Zitat) [6,59,72]. Recent evidence indicates that microbiota-driven type-I interferon signalling may promote immune dysfunction in chronic liver disease by inducing IL-10 production and thereby contributing to T-cell dysfunction [67]. In addition, bacterial DNA-induced type-I interferon signalling has been shown to shape peritoneal immunity during spontaneous bacterial peritonitis, including caspase-5-mediated progranulin release associated with adverse outcomes [73]. This dual phenotype—hyperinflammation together with defective pathogen clearance—is now considered a defining feature of infection susceptibility in decompensated cirrhosis [4,58].

Recent evidence highlights a role for type-I interferon signalling induced by bacterial DNA in promoting immune paralysis. This pathway drives IL-10 production, leading to T-cell dysfunction, and modulates peritoneal immunity during spontaneous bacterial peritonitis (SBP), including caspase-5-mediated progranulin release associated with adverse outcomes [67,73].

The combined disruption of epithelial, immune, and vascular barriers facilitates bacterial translocation, which represents a central pathogenic event in cirrhosis and underlies the development of spontaneous bacterial peritonitis (SBP). SBP arises when bacteria from the intestinal lumen cross the impaired barrier, disseminate via the portal circulation, and colonise ascitic fluid. The ascitic environment, characterised by reduced complement activity, impaired neutrophil function, and diminished opsonic capacity, allows bacterial proliferation even at low inocula [1,11,74].

The microbiology of SBP has evolved over time. While Gram-negative bacteria, particularly Escherichia coli and Klebsiella pneumoniae, remain predominant, Gram-positive organisms such as *Streptococcaceae* and *Enterococcaceae* now account for a substantial proportion of cases. In addition, multidrug-resistant pathogens are increasingly encountered, particularly in healthcare-associated infections, complicating empirical therapy [2,4,49]. This changing microbiological landscape further underscores that immune dysfunction in cirrhosis has direct therapeutic relevance, as impaired host defence now intersects with growing antimicrobial resistance [4].

Importantly, SBP is not merely a complication but a key driver of disease progression. Bacterial infections precipitate acute decompensation and frequently trigger acute-on-chronic liver failure, substantially increasing short-term mortality. The systemic inflammatory response exacerbates circulatory dysfunction, promotes organ failure, and reinforces a vicious cycle in which cirrhosis predisposes to infection, and infection accelerates disease progression [1,3].

Overall, the interplay between intestinal barrier dysfunction, microbial dysbiosis, and cirrhosis-associated immune dysfunction defines a central pathogenic axis in advanced liver disease. SBP represents a prototypical clinical manifestation of this process, illustrating how impaired barrier integrity and defective immune responses converge to drive severe infectious complications in cirrhosis.

## 4. Mechanisms of Barrier Failure in Liver Cirrhosis

**Figure 2:**
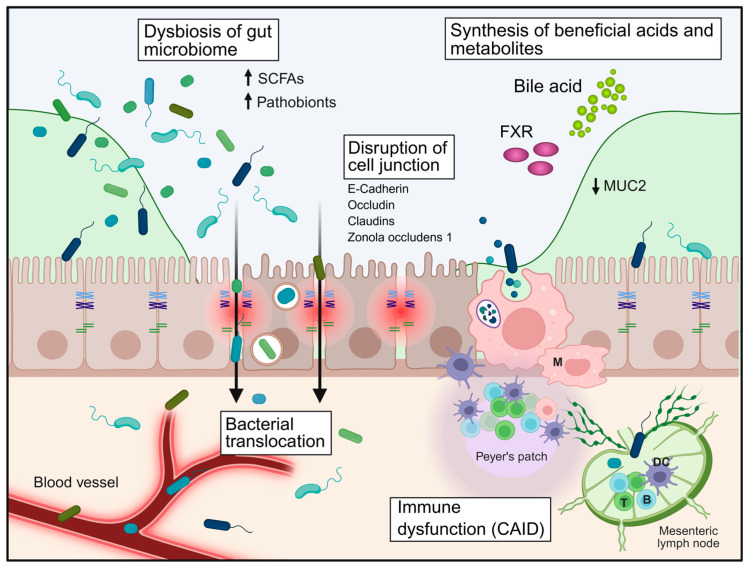
Gut microbiota dysbiosis-driven disruption of intestinal barrier integrity and immune dysfunction in liver cirrhosis. Schematic representation of the mechanisms by which alterations in the gut microbiome contribute to epithelial barrier breakdown and downstream immune activation. Dysbiosis is characterised by an imbalance in microbial communities, including an increase in pathobionts and altered production of microbial metabolites such as short-chain fatty acids (SCFAs). These changes impair epithelial homeostasis and promote disruption of intercellular junctional complexes, including E-cadherin, occludin, claudins, and zonula occludens-1 (ZO-1), resulting in increased intestinal permeability (“leaky gut”). Compromised barrier integrity facilitates bacterial translocation across the epithelium into the lamina propria via multiple routes, including paracellular passage through disrupted tight junctions, reduced mucus protection, and transcellular endocytic transport, and into the systemic circulation via the underlying vasculature. Concomitantly, dysbiosis alters bile acid metabolism and signalling through receptors such as the farnesoid X receptor (FXR), influencing the synthesis of beneficial metabolites and mucosal defence pathways. A reduction in mucin production (e.g., MUC2) weakens the mucus layer, further exposing epithelial cells to luminal microbes. Translocated bacteria and microbial products activate resident immune cells, including macrophages (M), dendritic cells (DC), and lymphocytes (T and B cells), particularly within gut-associated lymphoid structures such as Peyer’s patches and mesenteric lymph nodes. This leads to aberrant immune activation and chronic inflammation, contributing to conditions associated with immune dysregulation, such as cirrhosis-associated immune dysfunction (CAID). B, B-lymphocytes; MUC2, mucin 2; T, T-lymphocytes. Short upward black arrows indicate an increase; long downward black arrows indicate bacterial translocation. The figure was created in BioRender. Pollinger, K. (2026) https://BioRender.com/lln5uwf.

Barrier failure in liver cirrhosis is a multifactorial and progressive process that results from the convergence of epithelial, microbial, immune, vascular, and metabolic disturbances. Rather than representing a single defect, cirrhosis induces a coordinated breakdown of multiple, interconnected layers of the gastrointestinal barrier, including the mucus layer, epithelial junctional complexes, and the gut–vascular barrier (Figure 2). These processes reinforce each other and ultimately facilitate uncontrolled bacterial translocation, which underlies key complications such as spontaneous bacterial peritonitis (SBP) [1,4,9,62].

### 4.1. Hemodynamic and Structural Alterations: The Impact of Portal Hypertension

Portal hypertension is a major contributor to intestinal barrier dysfunction. Increased hydrostatic pressure within the splanchnic circulation leads to intestinal congestion, mucosal edema, and impaired microvascular perfusion, resulting in local hypoxia and altered epithelial metabolism. Hypoxia-induced signalling disrupts epithelial turnover and contributes to increased permeability [1,8,9,10].

Morphologically, portal hypertensive enteropathy is associated with villous blunting, epithelial apoptosis, and crypt distortion. However, experimental evidence demonstrates that portal hypertension alone is insufficient to induce the full spectrum of barrier dysfunction. In pre-hepatic portal hypertension models, mucus integrity, epithelial permeability, and bacterial translocation remain largely preserved, whereas cirrhotic models exhibit profound barrier impairment. These findings indicate that portal hypertension is an important but not sufficient driver of barrier failure on its own; its effects likely become more relevant in combination with cirrhosis-specific metabolic, microbial, and immune alterations. Overall, this highlights the importance of cirrhosis-specific factors in driving the full spectrum of barrier failure [5,8,10,32,75,76].

### 4.2. Epithelial Barrier Disruption and Tight Junction Remodelling

At the epithelial level, cirrhosis is characterised by structural and functional disruption of intercellular junctions. Key proteins, including claudins, occludin, zonula occludens (ZO) proteins, and E-cadherin, are downregulated, mislocalized, or degraded, resulting in loss of epithelial cohesion and increased paracellular permeability. Expression of key tight junction proteins, particularly occludin and claudin-1, declines with disease severity. Assimakopoulos et al. showed that both proteins were significantly reduced in compensated and decompensated cirrhosis compared with controls (*p* < 0.01), with more pronounced loss in decompensated disease (*p* < 0.05 vs. compensated cirrhosis) [31]. In addition, occludin and claudin-1 expression correlated inversely with Child–Pugh score, endotoxemia, and the grade of oesophageal varices. For occludin, loss of expression along the crypt–villus axis was especially marked in decompensated cirrhosis, with only 15 ± 8.9% positive enterocytes at the villous tip compared with 30 ± 9% in compensated cirrhosis and 100% in controls [31].

Mechanistically, epithelial disruption is driven by inflammatory cytokines, oxidative stress, and microbial products. In addition, proteolytic degradation of junctional proteins represents a critical mechanism of barrier failure. Both host-derived and bacterial proteases contribute to this process [9,31,32,77,78].

Importantly, bacteria isolated from patients with SBP, particularly *Escherichia coli* and *Proteus mirabilis*, directly induce junctional breakdown through two complementary mechanisms: enhanced ubiquitination and proteasomal degradation of occludin within epithelial cells, and bacterial protease-mediated cleavage of E-cadherin. These effects are dose- and time-dependent and are amplified by direct bacteria–epithelial cell interaction [9,79]. This establishes a feed-forward amplification loop in which translocating bacteria actively worsen the barrier defect that initially enabled their translocation [9,80]. Epithelial regeneration is further impaired by chronic inflammation and metabolic stress, disrupting the balance between proliferation and apoptosis and resulting in a sustained “leaky” epithelial barrier [9,32,81].

### 4.3. Mucus Layer Deterioration and Loss of Spatial Segregation

The mucus layer undergoes profound structural deterioration in cirrhosis, characterised by reduced thickness, goblet cell depletion, and decreased expression of the gel-forming mucin MUC2. Consequently, the normally sterile inner mucus layer becomes permeable, allowing bacterial colonisation in close proximity to the epithelium. Mechanistically, mucus barrier dysfunction is closely linked to altered bile acid metabolism and impaired FXR signalling, which regulate goblet cell differentiation and mucin production. Loss of FXR activity contributes directly to reduced MUC2 expression and mucus layer thinning [5,44,82,83].

In parallel, dysbiosis-associated depletion of short-chain fatty acids (SCFAs), particularly butyrate, further compromises mucus integrity by impairing epithelial metabolism and mucin synthesis. Loss of spatial compartmentalisation is a critical step in barrier failure, enabling direct microbial–epithelial interaction and promoting inflammatory activation [19,51,84,85].

### 4.4. Microbial Dysbiosis and Metabolic Perturbations

Cirrhosis is associated with profound alterations of the intestinal microbiome, characterised by reduced diversity, depletion of beneficial commensals, and expansion of pathobionts such as *Enterobacteriaceae*, *Enterococcaceae*, and *Streptococcaceae*. These compositional changes are accompanied by significant metabolic disturbances (Figure 2) [19,86,87,88].

A central consequence is reduced production of SCFAs, particularly butyrate, which serves as a key energy source for colonocytes and supports epithelial integrity. In cirrhosis, faecal butyrate levels are markedly reduced by up to 40–70% and inversely correlate with portal hypertension severity, endotoxemia, and systemic inflammation. SCFA depletion leads to impaired tight junction expression, reduced anti-inflammatory signalling, and diminished regulatory T-cell function. Experimental supplementation of butyrate restores tight junction proteins, reduces inflammatory cytokines, and limits bacterial translocation, underscoring its central role in barrier maintenance. Altered bile acid metabolism further contributes to dysbiosis and epithelial dysfunction by impairing FXR signalling, thereby linking metabolic and microbial disturbances [19,89,90,91].

### 4.5. Immune Dysregulation and Failure of Mucosal Defence

Barrier failure in cirrhosis is closely linked to CAID, which compromises both mucosal and systemic antimicrobial defence. Locally, reduced secretion of antimicrobial peptides and impaired IgA-mediated immune exclusion weaken the chemical barrier. The intestinal mucosa exhibits a pro-inflammatory immune profile driven by dysbiosis, including expansion of activated lymphocytes and a Th1-skewed response that paradoxically promotes barrier disruption [4,59].

Systemically, impaired hepatic immune surveillance plays a central role. Kupffer cells exhibit reduced phagocytic capacity, allowing gut-derived bacteria to escape hepatic clearance. In parallel, decreased hepatic synthesis of complement proteins compromises opsonisation and bactericidal activity, particularly within ascitic fluid [7,92,93,94].

Neutrophil dysfunction represents a hallmark of immune failure, characterised by impaired chemotaxis, reduced phagocytosis, and diminished intracellular killing capacity. At the same time, increased extracellular release of reactive oxygen species (ROS) contributes to oxidative stress and may further damage epithelial and endothelial barriers [95,96,97].

### 4.6. Gut–Vascular Barrier Dysfunction and Systemic Dissemination

Beyond epithelial disruption, the gut–vascular barrier (GVB) represents a critical checkpoint controlling bacterial entry into the circulation. In cirrhosis, the GVB is profoundly impaired, characterised by increased endothelial permeability and upregulation of plasmalemma vesicle-associated protein (PV1) [5,10,98].

Importantly, cirrhosis—but not portal hypertension alone—disrupts the GVB, allowing bacteria that cross the epithelium to access the portal circulation. Intestinal blood vessel-associated macrophages play a crucial protective role at this interface. In cirrhosis, these cells exhibit reduced bacterial clearance, impaired vascular interactions, and altered chemokine signalling. Their depletion results in bacterial translocation even in the absence of liver disease, underscoring their essential role in maintaining vascular barrier integrity [5,10]. In addition, portosystemic shunting allows bacteria and microbial products to bypass hepatic filtration, further facilitating systemic dissemination [1,4,5].

### 4.7. Integration of Barrier Failure and Link to SBP

These mechanisms are highly interconnected and mutually reinforcing. Portal hypertension promotes epithelial and vascular dysfunction, dysbiosis alters metabolic and immune signalling, and immune dysfunction further weakens barrier integrity. Together, these processes result in progressive loss of compartmentalisation between the intestinal lumen and the internal milieu [4,86,99,100]. This integrated, multi-layered failure—encompassing structural disruption, microbial imbalance, immune dysfunction, vascular permeability, and metabolic alterations—culminates in bacterial translocation, the central pathogenic event linking barrier dysfunction to clinical complications [9,10,32]. Importantly, this process is not unidirectional. Translocating bacteria may further exacerbate barrier dysfunction through direct interaction with epithelial cells. Bacterial proteases and host proteasome-dependent mechanisms contribute to the degradation of junctional proteins, thereby amplifying epithelial permeability in a feed-forward manner and perpetuating inflammatory activation [1,2,9].

In patients with cirrhosis and ascites, this process may culminate in spontaneous bacterial peritonitis, a prototypical manifestation of advanced barrier failure [9,32]. The self-amplifying nature of this system—where barrier dysfunction promotes bacterial translocation, and translocation further aggravates barrier disruption and systemic inflammation—explains the progressive increase in infection susceptibility with advancing cirrhosis [1,4,5].

From a therapeutic perspective, this integrated model highlights multiple potential intervention points. Strategies targeting the gut microbiome (e.g., probiotics, antibiotics, or faecal microbiota transplantation), restoration of barrier function (e.g., FXR agonists, bile acid modulation, or short-chain fatty acid supplementation), stabilisation of epithelial junctions through inhibition of bacterial or host proteases, and immunomodulatory approaches aimed at correcting cirrhosis-associated immune dysfunction may all contribute to reducing bacterial translocation. In addition, interventions that reduce portal hypertension may indirectly improve barrier integrity [5,19,51,101,102].

**Figure 3:**
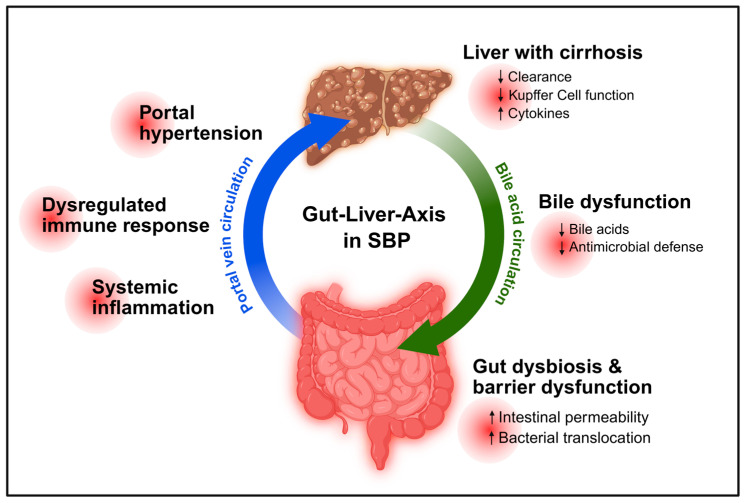
The gut–liver axis in cirrhosis and its contribution to spontaneous bacterial peritonitis (SBP). The gut–liver axis represents a dynamic, bidirectional communication system linking the intestinal microbiota and the liver via the portal circulation and bile acid signalling. In liver cirrhosis, this axis is profoundly disrupted. Alterations in bile acid synthesis and secretion impair intestinal antimicrobial defence and contribute to gut microbiota dysbiosis. Concurrently, structural and functional changes in the intestinal barrier increase permeability, facilitating bacterial translocation and the passage of microbial products into the portal circulation. Short upward arrows indicate an increase; short downward arrows indicate a decrease. The figure was created in BioRender. Pollinger, K. (2026) https://BioRender.com/lln5uwf.

Thus, barrier failure in cirrhosis should be understood as a dynamic and self-amplifying process that integrates structural, microbial, immune, vascular, and metabolic dysfunction. This framework not only explains the pathogenesis of spontaneous bacterial peritonitis but also provides a rationale for targeted therapeutic strategies aimed at restoring intestinal compartmentalisation and preventing infection-related complications (Figure 3).

## 5. Bacterial Translocation and SBP

Bacterial translocation represents the central pathogenic link between intestinal barrier dysfunction and infectious complications in liver cirrhosis (Figure 2). It is defined as the passage of viable bacteria or bacterial products from the intestinal lumen across the mucosal barrier to mesenteric lymph nodes and, subsequently, to the systemic circulation and extraintestinal sites. While low levels of translocation may occur under physiological conditions, this process is tightly controlled and does not result in clinically relevant infection. In cirrhosis, however, the coordinated breakdown of epithelial, immune, and vascular barriers leads to a pathological increase in both the frequency and magnitude of bacterial translocation [1,4,103].

### 5.1. Routes and Mechanisms of Bacterial Translocation

The paracellular route represents the predominant pathway, facilitated by disruption of tight junctions, thinning of the mucus layer, and loss of goblet cells with reduced MUC2 expression. These structural changes enable direct bacterial access to the epithelial surface. In addition, transcellular mechanisms—including endocytosis and uptake via specialised epithelial cells such as M cells—may contribute, particularly under conditions of severe barrier compromise. After crossing the epithelial barrier, microbes and microbial products may enter not only the portal circulation but also the intestinal lymphatic system, with mesenteric lymph nodes representing a key intermediate compartment in pathological bacterial translocation [5,31,77,104].

Importantly, bacterial translocation is not a passive process. Bacteria isolated from patients with spontaneous bacterial peritonitis actively disrupt epithelial integrity through dual mechanisms: bacterial proteases cleave E-cadherin, while bacteria exploit the host ubiquitin–proteasome system to degrade occludin. This establishes a feed-forward loop in which bacterial invasion further amplifies barrier dysfunction [1,9]. Beyond the epithelial layer, gut–vascular barrier dysfunction further facilitates bacterial access to the portal circulation [5,98].

### 5.2. Drivers of Increased Bacterial Translocation in Cirrhosis

Increased bacterial translocation in cirrhosis results from the interplay of structural barrier defects, microbial dysbiosis, immune dysfunction, and metabolic alterations. These factors act in a coordinated and self-reinforcing manner to promote both bacterial overgrowth and impaired containment of microbial invasion [4,5,10].

Microbial dysbiosis represents a central driver of translocation. Cirrhosis is associated with reduced microbial diversity and a shift toward a pro-inflammatory microbiota enriched in pathobionts such as *Enterobacteriaceae*, *Streptococcaceae*, and *Enterococcaceae*. At the same time, beneficial autochthonous taxa, particularly short-chain fatty acid (SCFA)-producing bacteria, are depleted. These compositional changes are not merely associative but functionally relevant, as bowel decontamination strategies have been shown to partially reverse dysbiosis and reduce bacterial translocation, supporting a causal relationship [4,19,87].

Immune dysregulation further amplifies this process. At the intestinal level, cirrhosis is associated with a pro-inflammatory immune phenotype characterised by Th1 polarisation, relative depletion of Th17 responses, and expansion of activated lymphocyte populations. These changes correlate with the degree of dysbiosis and markers of barrier dysfunction, such as faecal albumin loss. Despite this inflammatory milieu, antimicrobial defence is impaired, resulting in ineffective bacterial clearance [4,32,105].

CAID affects both mucosal and systemic compartments. Reduced secretion of antimicrobial peptides, impaired IgA-mediated immune exclusion, and dysfunctional macrophage activity weaken the first line of defence at the epithelial interface. Systemically, impaired Kupffer cell function and reduced complement activity limit bacterial clearance once translocation has occurred [4,32,106].

Metabolic alterations also play a critical role. Depletion of SCFAs, particularly butyrate, further weakens epithelial barrier integrity and mucosal immune regulation. In parallel, altered bile acid metabolism and reduced FXR signalling further compromise epithelial integrity and antimicrobial defence [4,19,24,89].

Together, these factors create a permissive environment in which bacterial overgrowth, barrier dysfunction, and impaired immune surveillance converge to promote pathological bacterial translocation in cirrhosis.

### 5.3. Spontaneous Bacterial Peritonitis: Pathogenesis

SBP represents the most clinically relevant consequence of pathological bacterial translocation in cirrhosis. It is defined as an infection of ascitic fluid in the absence of an intra-abdominal surgically treatable source and occurs predominantly in patients with advanced liver disease and ascites [1,11,107].

The pathogenesis of SBP involves a sequential process in which enteric bacteria translocate across the impaired intestinal barrier, reach mesenteric lymph nodes, and subsequently disseminate via the portal and systemic circulation. This process also involves the intestinal lymphatic system, which serves as an important route for the transport of bacteria and bacterial products from the gut to mesenteric lymph nodes before further systemic dissemination [9,32]. Due to impaired hepatic reticuloendothelial function and portosystemic shunting, these bacteria escape hepatic clearance and ultimately reach the peritoneal cavity [1,4]. The ascitic environment, characterised by reduced opsonic and bactericidal activity as a consequence of cirrhosis-associated immune dysfunction, provides favourable conditions for bacterial survival and proliferation [93,108,109].

SBP is typically monomicrobial, reflecting the translocation of a single dominant bacterial strain. Polymicrobial ascitic infection, in contrast, is uncommon in true SBP and should raise suspicion of secondary peritonitis or an underlying intra-abdominal source such as bowel perforation [110]. Historically, the causative organisms have predominantly been aerobic Gram-negative enteric bacteria, particularly *Escherichia coli* and *Klebsiella pneumoniae* [111,112,113,114]. However, the microbiological spectrum has evolved substantially in recent decades. Gram-positive organisms now account for a significant proportion of cases, especially in healthcare-associated and nosocomial infections, including *Enterococcus* species, *Streptococcus* species, and *Staphylococcus aureus* [4,113,115,116]. In parallel, multidrug-resistant (MDR) organisms have emerged as a major clinical concern, accounting for up to 35–50% of infections in certain settings [4,113,117].

The clinical presentation of SBP is often heterogeneous and may be subtle. While classic symptoms include abdominal pain, fever, and tenderness, many patients present with nonspecific features such as altered mental status, worsening ascites, or acute kidney injury. Notably, due to cirrhosis-associated immune dysfunction, a significant proportion of patients fail to mount a febrile response, which may delay diagnosis [1,2,11].

Given this variability, early and systematic diagnostic evaluation is essential. Diagnostic paracentesis is recommended in all patients with cirrhosis and ascites who are hospitalised or show signs of clinical deterioration. The diagnosis of SBP is established by an ascitic fluid polymorphonuclear (PMN) cell count ≥ 250 cells/mm^3^, which represents the most sensitive diagnostic criterion. Current clinical guidelines recommend prompt analysis of ascitic fluid, including cell count and microbiological culture, with inoculation into blood culture bottles at the bedside to maximise diagnostic yield [11,111].

SBP is a life-threatening complication associated with high short-term mortality, particularly in patients with concomitant renal dysfunction or infection with multidrug-resistant organisms. Importantly, SBP is not only a consequence of advanced cirrhosis but also a major driver of disease progression, frequently precipitating acute decompensation and acute-on-chronic liver failure [4,113,118,119].

Overall, SBP reflects the convergence of increased bacterial translocation, impaired host defence, and a permissive peritoneal environment. The evolving microbiological landscape, combined with often subtle clinical presentation, underscores the need for a high index of suspicion, early diagnosis, and timely initiation of appropriate therapy.

### 5.4. Systemic Consequences and Disease Progression

SBP is not merely an infectious complication of cirrhosis but a major driver of systemic deterioration and disease progression. Cirrhosis predisposes to SBP through impaired host defences and increased bacterial translocation, and once infection occurs, the host response is characterised by a dysregulated systemic inflammatory response syndrome (SIRS) that profoundly affects circulatory function and organ integrity [9,120].

The translocation of bacteria and bacterial products, such as lipopolysaccharide (LPS) and bacterial DNA, activates innate immune pathways and induces the release of pro-inflammatory cytokines, including tumour necrosis factor (TNF) and interleukin-6 (IL-6). This systemic inflammatory response exacerbates the hyperdynamic circulation typical of cirrhosis, promoting splanchnic vasodilation, reducing effective arterial blood volume, and activating neurohumoral systems such as the renin–angiotensin–aldosterone system (RAAS) and the sympathetic nervous system [1,2,4,101]. These hemodynamic changes contribute directly to organ dysfunction. Acute kidney injury, including hepatorenal syndrome, is a frequent and prognostically critical complication of SBP. In parallel, systemic inflammation promotes intrahepatic microvascular dysfunction, further increasing portal hypertension and impairing hepatic perfusion. The severity of this inflammatory response correlates closely with the extent of organ failure and short-term mortality [1,2,4,101].

Bacterial infections, including SBP, are among the leading triggers of acute decompensation and acute-on-chronic liver failure (ACLF). They can precipitate decompensation even in previously compensated patients and are a major determinant of hospitalisation and mortality. In ACLF, excessive systemic inflammation coexists with profound immune dysfunction, resulting in a state of high susceptibility to secondary infections and multi-organ failure [2,3,4]. At the same time, SBP further aggravates CAID. Persistent immune activation leads to functional exhaustion of immune cells and contributes to immune paralysis, thereby impairing bacterial clearance and predisposing to recurrent or secondary infections. This bidirectional relationship—where cirrhosis promotes infection and infection accelerates disease progression—creates a vicious cycle that significantly worsens prognosis [2,6,7].

From a clinical perspective, early recognition and prompt treatment of SBP are essential to interrupt this cycle. In addition, preventive strategies play a key role in high-risk patients. Antibiotic prophylaxis is recommended in selected populations, including patients with prior SBP, advanced cirrhosis with low ascitic protein and impaired liver or renal function, and those with acute gastrointestinal bleeding. While such strategies reduce SBP incidence and the risk of decompensation, they must be balanced against the increasing prevalence of multidrug-resistant organisms. Adjunctive therapies such as albumin are established in the acute management of SBP to reduce renal dysfunction and mortality, whereas microbiome-targeted interventions remain under investigation [2,4,11].

Overall, SBP represents a pivotal event in the natural history of cirrhosis, linking intestinal barrier failure to systemic inflammation, organ dysfunction, and disease progression. Its impact extends beyond infection itself, driving the transition to acute decompensation and ACLF and significantly contributing to short-term mortality.

### 5.5. Clinical Implications and Risk Stratification

The risk of bacterial translocation and SBP is closely linked to the severity of cirrhosis and the extent of barrier and immune dysfunction. Patients with advanced liver disease, particularly those with decompensated cirrhosis, ascites, low ascitic protein concentration (<1.5 g/dL), renal dysfunction, or hyponatremia, are at particularly high risk and are specifically recognised in current guidance as candidates for risk-adapted preventive strategies [2,11]. In addition to liver disease severity, microbiome-related factors contribute to risk stratification. Reduced microbial diversity and cirrhosis-associated dysbiosis, characterised by loss of beneficial autochthonous taxa and enrichment of pathobionts such as *Enterobacteriaceae* and *Enterococcaceae*, are associated with increased endotoxemia, systemic inflammation, and susceptibility to SBP and other infection-related complications. These observations support the concept that SBP risk reflects not only the presence of ascites but also the degree of gut barrier failure and immune dysregulation [2,4].

The clinical context further modifies the risk. Hospitalisation, invasive procedures, and prior antibiotic exposure are well-recognised contributors, particularly in patients with advanced disease. In addition, alcohol use, malnutrition, and diabetes further weaken host defences and favour bacterial overgrowth. The use of proton pump inhibitors has also been associated with an increased risk of SBP and should be reassessed carefully, especially when no clear indication persists [2,121,122]. From a prognostic perspective, outcomes in SBP are determined more by host factors and the intensity of systemic inflammation than by bacterial virulence alone. High MELD scores, renal dysfunction, hyponatremia, and the development of acute-on-chronic liver failure identify patients with particularly poor short-term outcomes. At the same time, multidrug-resistant infections further complicate management and worsen prognosis [2,4,11]. These considerations have direct clinical implications. Risk stratification helps identify patients who may benefit from primary or secondary antibiotic prophylaxis and underscores the need for antimicrobial stewardship amid rising multidrug resistance. Current guidance supports prophylaxis in selected high-risk groups, whereas albumin remains established in the acute management of SBP rather than for routine prevention. Microbiome-targeted and barrier-restoring strategies are promising but remain investigational [4,11].

Overall, clinical risk stratification in SBP should be understood as an integrated assessment of liver disease severity, ascitic fluid characteristics, immune dysfunction, microbial imbalance, and environmental exposures. This framework is essential for identifying patients at highest risk, guiding preventive strategies, and improving outcomes in advanced cirrhosis [2,4,11].

## 6. The Microbiome and Dysbiosis in Cirrhosis

The intestinal microbiome plays a central role in maintaining gastrointestinal barrier function and immune homeostasis. In cirrhosis, this balanced ecosystem is profoundly altered, a condition referred to as dysbiosis, which actively contributes to barrier dysfunction, immune dysregulation, and bacterial translocation [123,124].

Cirrhosis-associated dysbiosis is characterised by a marked reduction in microbial diversity, typically in the range of 30–60%, accompanied by a shift in community composition [19,49,87]. Beneficial autochthonous taxa, particularly SCFA-producing bacteria such as members of the *Lachnospiraceae* and *Ruminococcaceae* families, are depleted [19,49,125]. In parallel, there is an expansion of potentially pathogenic organisms, including *Enterobacteriaceae*, *Enterococcaceae*, and *Streptococcaceae*. This compositional shift is associated with increased endotoxemia and a pro-inflammatory milieu [2,4,49,125].

Beyond compositional changes, dysbiosis in cirrhosis is characterised by profound functional alterations. Functional dysbiosis in cirrhosis includes reduced production of SCFAs, particularly butyrate, which contributes to impaired barrier maintenance and a pro-inflammatory intestinal milieu [89,101,126,127].

Alterations in bile acid metabolism further contribute to microbiome dysfunction. Cirrhosis is associated with a reduction in secondary bile acids and impaired signalling through bile acid receptors such as the FXR and TGR5. Decreased intestinal FXR activation has been linked to reduced expression of key barrier components, including zonula occludens-1, occludin, and antimicrobial peptides. These findings position FXR signalling as an important regulatory axis linking microbial metabolism to epithelial and vascular barrier integrity [5,82,128].

The clinical relevance of dysbiosis is increasingly recognised. Reduced microbial diversity has been associated with a higher risk of complications such as hepatic encephalopathy and spontaneous bacterial peritonitis, with patients in the lowest diversity quartile exhibiting markedly increased risk. In addition, microbiome alterations correlate with disease severity scores, including MELD and Child–Pugh classification, and have been linked to short-term survival [1,2,19,129].

Dysbiosis in cirrhosis is both a consequence and a driver of disease progression. Portal hypertension, impaired intestinal motility, altered bile acid composition, and repeated antibiotic exposure all contribute to microbial imbalance. In turn, dysbiosis exacerbates barrier dysfunction, promotes bacterial translocation, and sustains systemic inflammation, thereby reinforcing the gut–liver axis as a self-amplifying pathogenic loop [2,101,130].

Therapeutic modulation of the microbiome has therefore emerged as an attractive strategy. Non-absorbable antibiotics such as rifaximin may reduce bacterial overgrowth and have shown benefits in selected clinical settings, although their impact on SBP prevention and overall survival remains inconsistent [131,132,133]. Probiotics have demonstrated efficacy in reducing hepatic encephalopathy but have not consistently improved infection-related outcomes [134]. Faecal microbiota transplantation has shown promising results in early-phase studies, particularly for hepatic encephalopathy, but remains investigational, with larger controlled trials required [2,135]. Overall, while microbiome-targeted therapies hold promise, their role in preventing bacterial translocation and SBP is not yet fully established [136,137].

## 7. Therapeutic Perspectives

For decades, gastrointestinal barrier–related complications have been managed primarily with systemic antibiotics aimed at reducing the intraluminal microbial burden. In recent years, however, there has been a shift toward approaches that focus on more precisely restoring barrier function and modulating the microbiome, largely in response to the emergence of MDR bacteria in cirrhosis-related infections [4]. The following therapeutic approaches should be interpreted according to their current level of evidence, ranging from established clinical practice to early clinical investigation and largely experimental or conceptual strategies.

Management of acute SBP continues to rely on prompt administration of third-generation cephalosporins. These are effective in most cases of community-acquired SBP, together with intravenous albumin as a critical adjunct therapy [11,74,138]. For prophylaxis, oral norfloxacin and ciprofloxacin have long served as the standard of care. However, recent retrospective data have raised concerns, observing higher rates of SBP recurrence in some patients receiving secondary prophylaxis compared to those who were not [11,74,139]. This finding is likely driven by the presence of quinolone-resistant bacteria and the collapse of phage-bacterial networks in the gut. Trimethoprim/sulfamethoxazole represents a possible alternative to fluoroquinolone-based prophylaxis, but current peer-reviewed evidence does not establish clear superiority, and adequately powered head-to-head trials are needed [140,141,142,143,144].

Although quinolone-based strategies pose certain challenges, recent evidence indicates that rifaximin has shown promising results and may represent a viable alternative for reducing the risk of complications associated with advanced decompensated cirrhosis in patients awaiting liver transplantation. Future studies are needed to confirm the long-term efficacy and safety for the prevention of specifically SBP. Nevertheless, the broad use of any antibiotic must be balanced against the escalating risk of developing multidrug-resistant infections, highlighting the need for alternative, non-antibiotic agents [131,132,133,145,146].

A promising emerging approach is the development of non-absorbable, engineered nanoporous carbon adsorbents, such as Yaq-001, which specifically target the translocation of LPS in the setting of cirrhotic dysbiosis. Currently in the phase 2 CARBALIVE-SAFETY clinical trial in decompensated cirrhosis (NCT03202498, accessed on 20 March 2026), this strategy has demonstrated a favourable impact on gut microbiome composition, with significant effects on ammonia levels, endotoxemia, and inflammation [147,148].

Microbiome-based interventions also represent an area of early clinical interest. Faecal microbiota transplantation (FMT) has shown early clinical benefits in hepatic encephalopathy by restoring microbial diversity and boosting short-chain fatty acid (SCFA) production, thereby reinforcing the intestinal epithelial barrier [2,56,135,149]. Debate persists over donor stool composition and the microbiome’s impact on host metabolism and immunity, alongside practical questions about optimal FMT protocols. Thus, larger controlled studies are needed [2,51,149]. Similarly, probiotics, prebiotics, and synbiotics may improve selected surrogate outcomes, particularly in hepatic encephalopathy, but evidence for prevention of bacterial translocation or SBP remains limited.

Alternatives to FMT are being investigated through synthetic microbial consortia, such as the eight-strain combination used in the phase 1b MARCO trial (NCT06871111, accessed on 20 March 2026), which aims to reestablish metabolite production in patients with profound dysbiosis [150].

A more experimental, precision therapy is CRISPR-guided microbiome editing. This approach enables the selective silencing of antibiotic resistance genes in pathogenic bacteria while preserving beneficial commensal taxa [151,152,153]. Conceptually, bacteriophage therapy can also be of use, as it offers a specific way to target and lyse pathogenic pathobionts, like cytolysin-producing Enterococcus faecalis, linked to high mortality in alcoholic hepatitis. Human trials are now necessary to assess the efficacy of phage therapy in cirrhosis [154]. These approaches remain largely in the preclinical stage and require robust trials to assess efficacy and safety.

Probiotics and prebiotics represent more established clinical tools for modulating the gut–liver axis. Probiotics offer therapeutic benefits by displacing urease-producing bacteria, improving barrier integrity. They also modulate immune responses through anti-inflammatory pathways like IL-10 and Treg cell expansion [155]. Current studies indicate that probiotics are effective in reducing symptoms and ammonia levels in patients with minimal encephalopathy, but with a modest impact on mortality and overall disease progression [134,156,157]. Prebiotics and symbiotics are commonly used to reduce bacterial translocation and have been shown to increase the expression of occludin, claudin-3, and ZO-1, thereby helping restore impaired tight junctions [158]. In patients with liver cirrhosis, lactulose is a key prebiotic for treating hepatic encephalopathy, as it promotes the growth of Bifidobacterium species and reduces the systemic absorption of ammonium salts [131,159]. Although lactulose may have a role in SBP through its ability to modulate the gut microbiota, no clinical trials are currently evaluating this, and no guidelines presently recommend its use for this indication.

Targeting bile acid signalling pathways represents another clinical and emerging therapeutic frontier. FXR agonists, such as obeticholic acid, have been shown in animal models to stabilise both the epithelial barrier and the GVB by reducing PV-1 expression and increasing tight junction protein synthesis [5,160,161]. However, there are still many concerns regarding the safety of OCA use [162]. Similarly, TGR5 agonists are currently being evaluated for their potential to enhance intestinal motility, thereby shortening transit time and reducing the opportunity for bacterial overgrowth [163]. Dual FXR/TGR5 agonists may eventually provide a comprehensive approach to both structural repair and physiological optimisation of the gut–liver axis (Figure 3) [164,165].

Maintenance of the intestinal barrier can also be supported through targeted nutritional strategies. Oral recombinant intestinal alkaline phosphatase (recIAP), a clinical-stage candidate, detoxifies luminal LPS and upregulates tight junction proteins such as ZO-1 and occludin. Studies indicate that recIAP prevents bacterial translocation and attenuates systemic inflammation by blocking LPS-TLR4, making it a promising candidate for preserving mucosal integrity in cirrhosis [166,167,168]. However, despite its clinical-stage development, evidence in cirrhosis remains limited, and confirmatory clinical trials are still needed. Supplementation with SCFAs such as butyrate also enhances barrier integrity and reduces inflammation in preclinical models [89,169]. However, more extensive clinical validation is required to confirm its therapeutic benefit in humans. Glutamine may represent another supportive nutritional strategy, as it serves as an important metabolic substrate for enterocytes and has been associated with improved intestinal barrier function and reduced permeability in experimental and clinical settings [170,171]. However, evidence specifically in cirrhosis remains limited, and its role in preventing bacterial translocation or SBP has not yet been established.

Ongoing early-stage research is evaluating the effectiveness of microRNAs, such as miR-320a, which inhibits *Escherichia coli*-induced intestinal barrier damage in ulcerative colitis. Whether this translates to cirrhosis-related bacterial translocation remains to be tested (Zitat) [51,172,173]. Taken together, these strategies differ substantially in translational maturity: recIAP is an early clinical-stage candidate, glutamine has limited supportive evidence, whereas butyrate/SCFA supplementation and miR-320a remain largely preclinical in the context of cirrhosis.

The management of gastrointestinal barrier dysfunction in liver cirrhosis is transitioning from non-selective bacterial decontamination to a more targeted ecological restoration strategy focused on the gut–liver axis. To reduce decompensation events, such as SBP, and improve survival, it will be essential to develop synergistic protocols that integrate structural barrier stabilisation, nutritional support, and precision microbial modulation.

At the hepatic level, cirrhosis is characterised by reduced clearance capacity, impaired Kupffer cell function, and an exaggerated yet ineffective inflammatory response, leading to increased systemic cytokine production. These changes promote a state of immune dysfunction that fails to control translocated bacteria adequately. The resulting feed-forward loop between gut dysbiosis, barrier dysfunction, and hepatic immune impairment drives systemic inflammation and portal hypertension. Collectively, disruption of the gut–liver axis is a central mechanism in cirrhosis and plays a key role in the pathogenesis of SBP by facilitating bacterial translocation and impairing host defence mechanisms.

## 8. Conclusions

The gastrointestinal barrier represents a central interface between the host and the intestinal microbiota, integrating structural, immune, microbial, and metabolic components to maintain intestinal homeostasis. In liver cirrhosis, this finely balanced system becomes progressively disrupted, resulting in a coordinated breakdown of epithelial integrity, mucus organisation, immune regulation, and vascular barrier function.

This multi-layered barrier failure facilitates pathological bacterial translocation, which constitutes a key pathogenic mechanism underlying spontaneous bacterial peritonitis (SBP) and other infection-related complications. Importantly, bacterial translocation is not merely a consequence of advanced disease but actively contributes to systemic inflammation, circulatory dysfunction, and disease progression. The resulting interplay between barrier dysfunction, microbial dysbiosis, and cirrhosis-associated immune dysfunction establishes a self-amplifying pathogenic loop that drives decompensation and increases short-term mortality.

Recent advances have significantly improved our understanding of the molecular mechanisms involved in barrier failure, including tight junction remodelling, mucus layer deterioration, gut–vascular barrier dysfunction, and impaired bile acid–FXR signalling. At the same time, the role of the intestinal microbiome has emerged as a central determinant of disease progression, linking metabolic alterations to immune dysregulation and barrier integrity.

From a clinical perspective, these insights underscore the importance of early identification of patients at risk for bacterial translocation and SBP. Risk stratification, timely diagnosis, and appropriate antimicrobial therapy remain essential components of current management. However, the increasing prevalence of multidrug-resistant organisms highlights the limitations of conventional approaches based solely on antibiotic strategies.

Future therapeutic concepts are therefore shifting toward a more integrated approach targeting the gut–liver axis (Figure 3). Strategies aimed at restoring barrier function, modulating the microbiome, and correcting immune dysfunction hold considerable promise. These include FXR-targeted therapies, microbiome-based interventions, and approaches designed to stabilise epithelial and vascular barrier integrity. At the same time, interpretation of the available evidence requires caution, as the literature is characterised by heterogeneous definitions of dysbiosis and intestinal barrier dysfunction, and many mechanistic insights are derived from experimental models that cannot be fully extrapolated to human cirrhosis. While many of these strategies remain investigational, they offer the potential to move beyond symptomatic treatment toward disease-modifying interventions. Further well-phenotyped clinical studies will be needed to validate translational relevance and to define which barrier-targeted approaches are most effective in specific stages of cirrhosis.

In summary, gastrointestinal barrier dysfunction represents a central and unifying mechanism in the pathogenesis of cirrhosis-related complications. A deeper mechanistic understanding of this process provides a framework for the development of targeted therapies aimed at restoring intestinal compartmentalisation, preventing bacterial translocation, and ultimately improving clinical outcomes in patients with liver cirrhosis.

## Data Availability

No new data were created or analyzed in this study. Data sharing is not applicable to this article.

## Abbreviations

ACLF, acute-on-chronic liver failure; AMPK, 5′ adenosine monophosphate-activated protein kinase; CAID, cirrhosis-associated immune dysfunction; CRISPR, clustered regularly interspaced short palindromic repeats; FMT, faecal microbiota transplantation FXR, farnesoid X receptor; GALT, gut-associated lymphoid tissue; GI, gastrointestinal; GVB, gut-vascular barrier; IgA, immunoglobulin A; IL-6, interleukin 6; IL-10, interleukin 10; LGR5+, leucine-rich repeat G-protein coupled receptor 5-positive; LPS, lipopolysaccharide; MDR, multidrug-resistant; MELD, model of end-stage liver disease; MUC2, mucin 2; OCA, obeticholic acid; PMN, polymorphonuclear; PV-1, plasmalemma vesicle-associated protein; RAAS, renin–angiotensin–aldosterone system; recIAP, recombinant intestinal alkaline phosphatase; RegIIIβ, regenerating islet-derived protein 3-beta; ROS, reactive oxygen species; SBP, spontaneous bacterial peritonitis; SCFA, short-chain fatty acid; SIRS, systemic inflammatory response syndrome; TGR5, Takeda G protein-coupled receptor 5; Treg, regulatory T cells; TNF-α, tumour necrosis factor-α; ZO, zonula occulens.

## References

[1] Ginès P., Krag A., Abraldes J.G., Solà E., Fabrellas N., Kamath P.S. (2021). Liver cirrhosis. Lancet.

[2] Bajaj J.S., Kamath P.S., Reddy K.R. (2021). The Evolving Challenge of Infections in Cirrhosis. N. Engl. J. Med..

[3] Bajaj J.S., O’Leary J.G., Lai J.C., Wong F., Long M.D., Wong R.J., Kamath P.S. (2022). Acute-on-Chronic Liver Failure Clinical Guidelines. Am. J. Gastroenterol..

[4] Piano S., Bunchorntavakul C., Marciano S., Rajender Reddy K. (2024). Infections in cirrhosis. Lancet Gastroenterol. Hepatol..

[5] Sorribas M., Jakob M.O., Yilmaz B., Li H., Stutz D., Noser Y., de Gottardi A., Moghadamrad S., Hassan M., Albillos A. (2019). FXR modulates the gut-vascular barrier by regulating the entry sites for bacterial translocation in experimental cirrhosis. J. Hepatol..

[6] McGettigan B., Hernandez-Tejero M., Malhi H., Shah V. (2025). Immune Dysfunction and Infection Risk in Advanced Liver Disease. Gastroenterology.

[7] Albillos A., Lario M., Álvarez-Mon M. (2014). Cirrhosis-associated immune dysfunction: Distinctive features and clinical relevance. J. Hepatol..

[8] Simbrunner B., Mandorfer M., Trauner M., Reiberger T. (2019). Gut-liver axis signaling in portal hypertension. World J. Gastroenterol..

[9] Haderer M., Neubert P., Rinner E., Scholtis A., Broncy L., Gschwendtner H., Kandulski A., Pavel V., Mehrl A., Brochhausen C. (2022). Novel pathomechanism for spontaneous bacterial peritonitis: Disruption of cell junctions by cellular and bacterial proteases. Gut.

[10] Smets L., Viola M.F., Boesch M., Raman J., van Melkebeke L., Nobis M., Flint E., Pajk N., Brescia P., Silvestri A. (2025). Intestinal blood vessel-associated macrophages and gut-vascular barrier dysfunction in cirrhosis. Gut.

[11] Biggins S.W., Angeli P., Garcia-Tsao G., Ginès P., Ling S.C., Nadim M.K., Wong F., Kim W.R. (2021). Diagnosis, Evaluation, and Management of Ascites, Spontaneous Bacterial Peritonitis and Hepatorenal Syndrome: 2021 Practice Guidance by the American Association for the Study of Liver Diseases. Hepatology.

[12] Pellegrini C., Fornai M., D’Antongiovanni V., Antonioli L., Bernardini N., Derkinderen P. (2023). The intestinal barrier in disorders of the central nervous system. Lancet Gastroenterol. Hepatol..

[13] Di Sabatino A., Santacroce G., Rossi C.M., Broglio G., Lenti M.V. (2023). Role of mucosal immunity and epithelial-vascular barrier in modulating gut homeostasis. Intern. Emerg. Med..

[14] Neurath M.F., Artis D., Becker C. (2025). The intestinal barrier: A pivotal role in health, inflammation, and cancer. Lancet Gastroenterol. Hepatol..

[15] Breugelmans T., Oosterlinck B., Arras W., Ceuleers H., de Man J., Hold G.L., de Winter B.Y., Smet A. (2022). The role of mucins in gastrointestinal barrier function during health and disease. Lancet Gastroenterol. Hepatol..

[16] Pelaseyed T., Bergström J.H., Gustafsson J.K., Ermund A., Birchenough G.M.H., Schütte A., van der Post S., Svensson F., Rodríguez-Piñeiro A.M., Nyström E.E.L. (2014). The mucus and mucins of the goblet cells and enterocytes provide the first defense line of the gastrointestinal tract and interact with the immune system. Immunol. Rev..

[17] Chang J.T. (2020). Pathophysiology of Inflammatory Bowel Diseases. N. Engl. J. Med..

[18] Iftekhar A., Sigal M. (2021). Defence and adaptation mechanisms of the intestinal epithelium upon infection. Int. J. Med. Microbiol..

[19] Chang L., Liu Y., Li H., Yan J., Wu W., Chen N., Ma C., Zhao X., Chen J., Zhang J. (2025). Gut microbiome and its metabolites in liver cirrhosis: Mechanisms and clinical implications. Front. Cell. Infect. Microbiol..

[20] Kuo W.-T., Odenwald M.A., Turner J.R., Zuo L. (2022). Tight junction proteins occludin and ZO-1 as regulators of epithelial proliferation and survival. Ann. N. Y. Acad. Sci..

[21] Suzuki T. (2013). Regulation of intestinal epithelial permeability by tight junctions. Cell. Mol. Life Sci..

[22] Zhang K., Hornef M.W., Dupont A. (2015). The intestinal epithelium as guardian of gut barrier integrity. Cell. Microbiol..

[23] Gehart H., Clevers H. (2019). Tales from the crypt: New insights into intestinal stem cells. Nat. Rev. Gastroenterol. Hepatol..

[24] Iebba V., Guerrieri F., Di Gregorio V., Levrero M., Gagliardi A., Santangelo F., Sobolev A.P., Circi S., Giannelli V., Mannina L. (2018). Combining amplicon sequencing and metabolomics in cirrhotic patients highlights distinctive microbiota features involved in bacterial translocation, systemic inflammation and hepatic encephalopathy. Sci. Rep..

[25] Horowitz A., Chanez-Paredes S.D., Haest X., Turner J.R. (2023). Paracellular permeability and tight junction regulation in gut health and disease. Nat. Rev. Gastroenterol. Hepatol..

[26] Günzel D., Yu A.S.L. (2013). Claudins and the modulation of tight junction permeability. Physiol. Rev..

[27] Le S., Weber C.R., Raleigh D.R., Yu D., Turner J.R. (2011). Tight junction pore and leak pathways: A dynamic duo. Annu. Rev. Physiol..

[28] Jin Y., Blikslager A.T. (2020). The Regulation of Intestinal Mucosal Barrier by Myosin Light Chain Kinase/Rho Kinases. Int. J. Mol. Sci..

[29] Lynn K.S., Peterson R.J., Koval M. (2020). Ruffles and spikes: Control of tight junction morphology and permeability by claudins. Biochim. Biophys. Acta Biomembr..

[30] Le S. (2012). Tight junctions on the move: Molecular mechanisms for epithelial barrier regulation. Ann. N. Y. Acad. Sci..

[31] Assimakopoulos S.F., Tsamandas A.C., Tsiaoussis G.I., Karatza E., Triantos C., Vagianos C.E., Spiliopoulou I., Kaltezioti V., Charonis A., Nikolopoulou V.N. (2012). Altered intestinal tight junctions’ expression in patients with liver cirrhosis: A pathogenetic mechanism of intestinal hyperpermeability. Eur. J. Clin. Investig..

[32] Muñoz L., Borrero M.-J., Úbeda M., Conde E., Del Campo R., Rodríguez-Serrano M., Lario M., Sánchez-Díaz A.-M., Pastor O., Díaz D. (2019). Intestinal Immune Dysregulation Driven by Dysbiosis Promotes Barrier Disruption and Bacterial Translocation in Rats With Cirrhosis. Hepatology.

[33] Du Plessis J., Vanheel H., Janssen C.E.I., Roos L., Slavik T., Stivaktas P.I., Nieuwoudt M., van Wyk S.G., Vieira W., Pretorius E. (2013). Activated intestinal macrophages in patients with cirrhosis release NO and IL-6 that may disrupt intestinal barrier function. J. Hepatol..

[34] Johansson M.E.V., Sjövall H., Hansson G.C. (2013). The gastrointestinal mucus system in health and disease. Nat. Rev. Gastroenterol. Hepatol..

[35] Tonetti F.R., Eguileor A., Llorente C. (2024). Goblet cells: Guardians of gut immunity and their role in gastrointestinal diseases. eGastroenterology.

[36] Okumura R., Takeda K. (2024). The role of the mucosal barrier system in maintaining gut symbiosis to prevent intestinal inflammation. Semin. Immunopathol..

[37] Cheng H., Li H., Li Z., Wang Y., Liu L., Wang J., Ma X., Tan B. (2025). The role of glycosylated mucins in maintaining intestinal homeostasis and gut health. Anim. Nutr. (Zhongguo Xu Mu Shou Yi Xue Hui).

[38] Johansson M.E.V., Larsson J.M.H., Hansson G.C. (2011). The two mucus layers of colon are organized by the MUC2 mucin, whereas the outer layer is a legislator of host-microbial interactions. Proc. Natl. Acad. Sci. USA.

[39] Bergstrom K., Xia L. (2022). The barrier and beyond: Roles of intestinal mucus and mucin-type O-glycosylation in resistance and tolerance defense strategies guiding host-microbe symbiosis. Gut Microbes.

[40] Okumura R., Takeda K. (2017). Roles of intestinal epithelial cells in the maintenance of gut homeostasis. Exp. Mol. Med..

[41] Hockenberry A., Slack E., Stadtmueller B.M. (2023). License to Clump: Secretory IgA Structure-Function Relationships Across Scales. Annu. Rev. Microbiol..

[42] Mantis N.J., Rol N., Corthésy B. (2011). Secretory IgA’s complex roles in immunity and mucosal homeostasis in the gut. Mucosal Immunol..

[43] Song G., Xie Y., Yi L., Cheng W., Jia H., Shi W., Liu Q., Fang L., Xue S., Liu D. (2025). Bile acids affect intestinal barrier function through FXR and TGR5. Front. Med..

[44] Simbrunner B., Hofer B.S., Schwabl P., Zinober K., Petrenko O., Fuchs C., Semmler G., Marculescu R., Mandorfer M., Datz C. (2024). FXR-FGF19 signaling in the gut-liver axis is dysregulated in patients with cirrhosis and correlates with impaired intestinal defence. Hepatol. Int..

[45] Costa D., Trebicka J., Ripoll C., Moreau R., Jalan R., Reiberger T. (2025). Interaction of inflammation and portal hypertension in cirrhosis progression. Nat. Rev. Gastroenterol. Hepatol..

[46] Fuchs C.D., Simbrunner B., Baumgartner M., Campbell C., Reiberger T., Trauner M. (2025). Bile acid metabolism and signalling in liver disease. J. Hepatol..

[47] Verbeke L., Farre R., Verbinnen B., Covens K., Vanuytsel T., Verhaegen J., Komuta M., Roskams T., Chatterjee S., Annaert P. (2015). The FXR agonist obeticholic acid prevents gut barrier dysfunction and bacterial translocation in cholestatic rats. Am. J. Pathol..

[48] Wu Z., Zhou H., Liu D., Deng F. (2023). Alterations in the gut microbiota and the efficacy of adjuvant probiotic therapy in liver cirrhosis. Front. Cell. Infect. Microbiol..

[49] Liu Y., Chen Z., Li C., Sun T., Luo X., Jiang B., Liu M., Wang Q., Li T., Cao J. (2025). Associations between changes in the gut microbiota and liver cirrhosis: A systematic review and meta-analysis. BMC Gastroenterol..

[50] Wang Q., Chen C., Zuo S., Cao K., Li H. (2023). Integrative analysis of the gut microbiota and faecal and serum short-chain fatty acids and tryptophan metabolites in patients with cirrhosis and hepatic encephalopathy. J. Transl. Med..

[51] Tranah T.H., Edwards L.A., Schnabl B., Shawcross D.L. (2021). Targeting the gut-liver-immune axis to treat cirrhosis. Gut.

[52] Qiao Y., He C., Xia Y., Ocansey D.K.W., Mao F. (2025). Intestinal mucus barrier: A potential therapeutic target for IBD. Autoimmun. Rev..

[53] Tingler A.M., Engevik M.A. (2025). Breaking down barriers: Is intestinal mucus degradation by Akkermansia muciniphila beneficial or harmful?. Infect. Immun..

[54] Almeqdadi M., Gordon F.D. (2024). Farnesoid X Receptor Agonists: A Promising Therapeutic Strategy for Gastrointestinal Diseases. Gastro Hep Adv..

[55] Bajaj J.S., Ng S.C., Schnabl B. (2022). Promises of microbiome-based therapies. J. Hepatol..

[56] Bajaj J.S., Salzman N.H., Acharya C., Sterling R.K., White M.B., Gavis E.A., Fagan A., Hayward M., Holtz M.L., Matherly S. (2019). Fecal Microbial Transplant Capsules Are Safe in Hepatic Encephalopathy: A Phase 1, Randomized, Placebo-Controlled Trial. Hepatology.

[57] Bloom P.P., Tapper E.B. (2023). Lactulose in cirrhosis: Current understanding of efficacy, mechanism, and practical considerations. Hepatol. Commun..

[58] Rodríguez-Negrete E.V., Gálvez-Martínez M., Sánchez-Reyes K., Fajardo-Felix C.F., Pérez-Reséndiz K.E., Madrigal-Santillán E.O., Morales-González Á., Morales-González J.A. (2024). Liver Cirrhosis: The Immunocompromised State. J. Clin. Med..

[59] Albillos A., Martin-Mateos R., van der Merwe S., Wiest R., Jalan R., Álvarez-Mon M. (2022). Cirrhosis-associated immune dysfunction. Nat. Rev. Gastroenterol. Hepatol..

[60] Yu S., Gao N. (2015). Compartmentalizing intestinal epithelial cell toll-like receptors for immune surveillance. Cell. Mol. Life Sci..

[61] Owen A.M., Luan L., Burelbach K.R., McBride M.A., Stothers C.L., Boykin O.A., Sivanesam K., Schaedel J.F., Patil T.K., Wang J. (2022). MyD88-dependent signaling drives toll-like receptor-induced trained immunity in macrophages. Front. Immunol..

[62] Kawai T., Akira S. (2010). The role of pattern-recognition receptors in innate immunity: Update on Toll-like receptors. Nat. Immunol..

[63] Fan Y., Li Y., Chu Y., Liu J., Cui L., Zhang D. (2021). Toll-Like Receptors Recognize Intestinal Microbes in Liver Cirrhosis. Front. Immunol..

[64] Zhou Y., Yu S., Zhang W. (2023). NOD-like Receptor Signaling Pathway in Gastrointestinal Inflammatory Diseases and Cancers. Int. J. Mol. Sci..

[65] He Y., Hara H., Núñez G. (2016). Mechanism and Regulation of NLRP3 Inflammasome Activation. Trends Biochem. Sci..

[66] Lu Q., Hitch T.C.A., Zhou J.Y., Dwidar M., Sangwan N., Lawrence D., Nolan L.S., Espenschied S.T., Newhall K.P., Han Y. (2024). A host-adapted auxotrophic gut symbiont induces mucosal immunodeficiency. Science.

[67] Hackstein C.-P., Spitzer J., Symeonidis K., Horvatic H., Bedke T., Steglich B., Klein S., Assmus L.M., Odainic A., Szlapa J. (2023). Interferon-induced IL-10 drives systemic T-cell dysfunction during chronic liver injury. J. Hepatol..

[68] van der Merwe S., Chokshi S., Bernsmeier C., Albillos A. (2021). The multifactorial mechanisms of bacterial infection in decompensated cirrhosis. J. Hepatol..

[69] Ohlendorf V., Buttler L., Maasoumy B. (2026). Leberzirrhose-assoziierte Immundysfunktion (CAID). Dtsch. Med. Wochenschr..

[70] Jenne C.N., Kubes P. (2013). Immune surveillance by the liver. Nat. Immunol..

[71] Rimola A., Soto R., Bory F., Arroyo V., Piera C., Rodes J. (1984). Reticuloendothelial system phagocytic activity in cirrhosis and its relation to bacterial infections and prognosis. Hepatology.

[72] Seitz R., Müller M., Gülow K. (2026). Extracellular Redox Balance as a Determinant of Immune Regulation and Tissue Inflammation. Antioxidants.

[73] Rooney M., Duduskar S.N., Ghait M., Reißing J., Stengel S., Reuken P.A., Quickert S., Zipprich A., Bauer M., Russo A.J. (2024). Type-I interferon shapes peritoneal immunity in cirrhosis and drives caspase-5-mediated progranulin release upon infection. J. Hepatol..

[74] Angeli P., Bernardi M., Villanueva C., Francoz C., Mookerjee R.P., Trebicka J., Krag A., Laleman W., Gines P. (2018). EASL Clinical Practice Guidelines for the management of patients with decompensated cirrhosis. J. Hepatol..

[75] Ponziani F.R., Zocco M.A., Cerrito L., Gasbarrini A., Pompili M. (2018). Bacterial translocation in patients with liver cirrhosis: Physiology, clinical consequences, and practical implications. Expert Rev. Gastroenterol. Hepatol..

[76] Tsiaoussis G.I., Papaioannou E.C., Kourea E.P., Assimakopoulos S.F., Theocharis G.I., Petropoulos M., Theopistos V.I., Diamantopoulou G.G., Lygerou Z., Spiliopoulou I. (2018). Expression of α-Defensins, CD20+ B-lymphocytes, and Intraepithelial CD3+ T-lymphocytes in the Intestinal Mucosa of Patients with Liver Cirrhosis: Emerging Mediators of Intestinal Barrier Function. Dig. Dis. Sci..

[77] Pijls K.E., Jonkers D.M.A.E., Elamin E.E., Masclee A.A.M., Koek G.H. (2013). Intestinal epithelial barrier function in liver cirrhosis: An extensive review of the literature. Liver Int..

[78] Fondevila M.F., Kreimeyer H., Hsu C.L., Tamargo-Azpilicueta J., Le Day Z., Gritsenko M., Attah K., Cabré N., Harberts A., Tonetti F.R. (2026). Macrophage-derived cathepsin B disrupts intestinal tight junctions through occludin degradation and promotes alcohol-associated liver disease. J. Hepatol..

[79] Devaux C.A., Mezouar S., Mege J.-L. (2019). The E-Cadherin Cleavage Associated to Pathogenic Bacteria Infections Can Favor Bacterial Invasion and Transmigration, Dysregulation of the Immune Response and Cancer Induction in Humans. Front. Microbiol..

[80] Huber P. (2020). Targeting of the apical junctional complex by bacterial pathogens. Biochim. Biophys. Acta Biomembr..

[81] Rogers A.P., Mileto S.J., Lyras D. (2023). Impact of enteric bacterial infections at and beyond the epithelial barrier. Nat. Rev. Microbiol..

[82] Cai Y., Chen X., Wang H., Hou L., Zheng R., Wang Y., Jiang W., Tang W. (2025). Investigating intestinal farnesoid X receptor functions at the intestinal mucosal barrier and in the intestinal microbiota in a biliary obstruction mouse model. Am. J. Physiol. Gastrointest. Liver Physiol..

[83] Distrutti E., Santucci L., Cipriani S., Renga B., Schiaroli E., Ricci P., Donini A., Fiorucci S. (2015). Bile acid activated receptors are targets for regulation of integrity of gastrointestinal mucosa. J. Gastroenterol..

[84] Jin M., Kalainy S., Baskota N., Chiang D., Deehan E.C., McDougall C., Tandon P., Martínez I., Cervera C., Walter J. (2019). Faecal microbiota from patients with cirrhosis has a low capacity to ferment non-digestible carbohydrates into short-chain fatty acids. Liver Int..

[85] Paone P., Cani P.D. (2020). Mucus barrier, mucins and gut microbiota: The expected slimy partners?. Gut.

[86] Haidar G., Singh N. (2021). The Evolving Challenge of Infections in Cirrhosis. N. Engl. J. Med..

[87] Solé C., Guilly S., Da Silva K., Llopis M., Le-Chatelier E., Huelin P., Carol M., Moreira R., Fabrellas N., de Prada G. (2021). Alterations in Gut Microbiome in Cirrhosis as Assessed by Quantitative Metagenomics: Relationship With Acute-on-Chronic Liver Failure and Prognosis. Gastroenterology.

[88] Ma C., Yang J., Fu X.-N., Luo J.-Y., Liu P., Zeng X.-L., Li X.-Y., Zhang S.-L., Zheng S. (2025). Microbial characteristics of gut microbiome dysbiosis in patients with chronic liver disease. World J. Hepatol..

[89] Juanola O., Ferrusquía-Acosta J., García-Villalba R., Zapater P., Magaz M., Marín A., Olivas P., Baiges A., Bellot P., Turon F. (2019). Circulating levels of butyrate are inversely related to portal hypertension, endotoxemia, and systemic inflammation in patients with cirrhosis. FASEB J..

[90] Zhu F., Zheng S., Zhao M., Shi F., Zheng L., Wang H. (2023). The regulatory role of bile acid microbiota in the progression of liver cirrhosis. Front. Pharmacol..

[91] Simbrunner B., Trauner M., Reiberger T. (2021). Review article: Therapeutic aspects of bile acid signalling in the gut-liver axis. Aliment. Pharmacol. Ther..

[92] Pose E., Coll M., Martínez-Sánchez C., Zeng Z., Surewaard B.G.J., Català C., Velasco-de Andrés M., Lozano J.J., Ariño S., Fuster D. (2021). Programmed Death Ligand 1 Is Overexpressed in Liver Macrophages in Chronic Liver Diseases, and Its Blockade Improves the Antibacterial Activity Against Infections. Hepatology.

[93] Akalin H.E., Laleli Y., Telatar H. (1983). Bactericidal and opsonic activity of ascitic fluid from cirrhotic and noncirrhotic patients. J. Infect. Dis..

[94] Runyon B.A., Morrissey R.L., Hoefs J.C., Wyle F.A. (1985). Opsonic activity of human ascitic fluid: A potentially important protective mechanism against spontaneous bacterial peritonitis. Hepatology.

[95] Taylor N.J., Manakkat Vijay G.K., Abeles R.D., Auzinger G., Bernal W., Ma Y., Wendon J.A., Shawcross D.L. (2014). The severity of circulating neutrophil dysfunction in patients with cirrhosis is associated with 90-day and 1-year mortality. Aliment. Pharmacol. Ther..

[96] Rajkovic I.A., Williams R. (1986). Abnormalities of neutrophil phagocytosis, intracellular killing and metabolic activity in alcoholic cirrhosis and hepatitis. Hepatology.

[97] Moreau R., Périanin A., Arroyo V. (2019). Review of Defective NADPH Oxidase Activity and Myeloperoxidase Release in Neutrophils From Patients With Cirrhosis. Front. Immunol..

[98] Haedge F., Reuken P.A., Reißing J., Große K., Frissen M., El-Hassani M., Aschenbach R., Teichgräber U., Stallmach A., Bruns T. (2025). Surrogate Markers of Intestinal Permeability, Bacterial Translocation and Gut-Vascular Barrier Damage Across Stages of Cirrhosis. Liver Int..

[99] Nie G., Zhang H., Xie D., Yan J., Li X. (2023). Liver cirrhosis and complications from the perspective of dysbiosis. Front. Med..

[100] Guan H., Zhang X., Kuang M., Yu J. (2022). The gut-liver axis in immune remodeling of hepatic cirrhosis. Front. Immunol..

[101] Albillos A., de Gottardi A., Rescigno M. (2020). The gut-liver axis in liver disease: Pathophysiological basis for therapy. J. Hepatol..

[102] Zhou H., Huang Y., Chen C., Song M., Hylemon P.B. (2026). Gut microbiome and bile acid metabolism in liver disease: Mechanisms, clinical implications, and therapeutic opportunities. Pharmacol. Rev..

[103] Bellot P., Francés R., Such J. (2013). Pathological bacterial translocation in cirrhosis: Pathophysiology, diagnosis and clinical implications. Liver Int..

[104] Zhang L., Tai Y., Tang S., Zhao C., Tong H., Gao J., Tang C. (2021). Compromised Ileal Mucus Barrier Due to Impaired Epithelial Homeostasis Caused by Notch1 Signaling in Cirrhotic Rats. Dig. Dis. Sci..

[105] Shi H., Lv L., Cao H., Lu H., Zhou N., Yang J., Jiang H., Dong H., Hu X., Yu W. (2017). Bacterial translocation aggravates CCl4-induced liver cirrhosis by regulating CD4+ T cells in rats. Sci. Rep..

[106] Bernsmeier C., van der Merwe S., Périanin A. (2020). Innate immune cells in cirrhosis. J. Hepatol..

[107] Ginès P., Cárdenas A., Arroyo V., Rodés J. (2004). Management of cirrhosis and ascites. N. Engl. J. Med..

[108] Ibidapo-Obe O., Rooney M.D., Bruns T. (2025). Peritoneal immunity in decompensated cirrhosis. Expert Rev. Clin. Immunol..

[109] Nieto J.C., Perea L., Soriano G., Zamora C., Cantó E., Medina A., Poca M., Sanchez E., Roman E., Julià G. (2018). Ascitic fluid regulates the local innate immune response of patients with cirrhosis. J. Leukoc. Biol..

[110] Soriano G., Castellote J., Alvarez C., Girbau A., Gordillo J., Baliellas C., Casas M., Pons C., Román E.M., Maisterra S. (2010). Secondary bacterial peritonitis in cirrhosis: A retrospective study of clinical and analytical characteristics, diagnosis and management. J. Hepatol..

[111] Miller J.M., Binnicker M.J., Campbell S., Carroll K.C., Chapin K.C., Gonzalez M.D., Harrington A., Jerris R.C., Kehl S.C., Leal S.M. (2024). Guide to Utilization of the Microbiology Laboratory for Diagnosis of Infectious Diseases: 2024 Update by the Infectious Diseases Society of America (IDSA) and the American Society for Microbiology (ASM). Clin. Infect. Dis..

[112] Furey C., Zhou S., Park J.H., Foong A., Chowdhury A., Dawit L., Lee V., Vergara-Lluri M., She R., Kahn J. (2023). Impact of Bacteria Types on the Clinical Outcomes of Spontaneous Bacterial Peritonitis. Dig. Dis. Sci..

[113] Zhang X., Li X.-X., Song J.-W., Zhang X.-C., Zhen C., Bi J.-F., Lu F.-Y., Chen S.-M., Dan Huo D., Zhao P. (2023). Clinical features, microbial spectrum, and antibiotic susceptibility patterns of spontaneous bacterial peritonitis in cirrhotic patients. Dig. Liver Dis..

[114] Blanchard F., Henry B., Vijayaratnam S., Canouï E., Moura A., Thouvenot P., Bracq-Dieye H., Tessaud-Rita N., Valès G., Diakité A. (2024). Listeria monocytogenes-associated spontaneous bacterial peritonitis in France: A nationwide observational study of 208 cases. Lancet Infect. Dis..

[115] Kitchen P., Schütte S.L., Tergast T.L., Maasoumy B., Braun L., Esche U.V.d., Häcker G., Mücke M.M., Reincke M., Schultheiss M. (2026). Altered Pathogen Spectrum of Spontaneous Bacterial Peritonitis in Patients Treated With Proton Pump Inhibitors. Aliment. Pharmacol. Ther..

[116] Alexopoulou A., Papadopoulos N., Eliopoulos D.G., Alexaki A., Tsiriga A., Toutouza M., Pectasides D. (2013). Increasing frequency of gram-positive cocci and gram-negative multidrug-resistant bacteria in spontaneous bacterial peritonitis. Liver Int..

[117] Li H., Wieser A., Zhang J., Liss I., Markwardt D., Hornung R., Neumann-Cip A.C., Mayerle J., Gerbes A., Steib C.J. (2020). Patients with cirrhosis and SBP: Increase in multidrug-resistant organisms and complications. Eur. J. Clin. Investig..

[118] Hung T.-H., Wang C.-Y., Tsai C.-C., Lee H.-F. (2024). Short and long-term mortality of spontaneous bacterial peritonitis in cirrhotic patients. Medicine.

[119] Ruiz-del-Arbol L., Urman J., Fernández J., González M., Navasa M., Monescillo A., Albillos A., Jiménez W., Arroyo V. (2003). Systemic, renal, and hepatic hemodynamic derangement in cirrhotic patients with spontaneous bacterial peritonitis. Hepatology.

[120] Simbrunner B., Caparrós E., Neuwirth T., Schwabl P., Königshofer P., Bauer D., Marculescu R., Trauner M., Scheiner B., Stary G. (2023). Bacterial translocation occurs early in cirrhosis and triggers a selective inflammatory response. Hepatol. Int..

[121] Xu H.B., Wang H.D., Li C.H., Ye S., Dong M.S., Xia Q.J., Zhang A.Q., Pan K., Ge X.L., Dong J.H. (2015). Proton pump inhibitor use and risk of spontaneous bacterial peritonitis in cirrhotic patients: A systematic review and meta-analysis. Genet. Mol. Res..

[122] El-Azab G. (2024). Proton Pump Inhibitors in Patients with Cirrhosis: Pharmacokinetics, Benefits and Drawbacks. Curr. Gastroenterol. Rep..

[123] Philips C.A., Augustine P. (2022). Gut Barrier and Microbiota in Cirrhosis. J. Clin. Exp. Hepatol..

[124] Chopyk D.M., Grakoui A. (2020). Contribution of the Intestinal Microbiome and Gut Barrier to Hepatic Disorders. Gastroenterology.

[125] Chen Y., Yang F., Lu H., Wang B., Chen Y., Lei D., Wang Y., Zhu B., Li L. (2011). Characterization of fecal microbial communities in patients with liver cirrhosis. Hepatology.

[126] Qin N., Yang F., Li A., Prifti E., Chen Y., Shao L., Guo J., Le Chatelier E., Yao J., Wu L. (2014). Alterations of the human gut microbiome in liver cirrhosis. Nature.

[127] Peng L., Li Z.-R., Green R.S., Holzman I.R., Lin J. (2009). Butyrate enhances the intestinal barrier by facilitating tight junction assembly via activation of AMP-activated protein kinase in Caco-2 cell monolayers. J. Nutr..

[128] Qian S., Su Z., Lin J., Hou Q., Wang X., Li Y., Wang J., Huang C., Wang Z., Cubero F.J. (2025). Inhibition of Farnesoid-x-receptor signaling during abdominal sepsis by dysbiosis exacerbates gut barrier dysfunction. Cell Commun. Signal..

[129] Bajaj J.S., Heuman D.M., Hylemon P.B., Sanyal A.J., White M.B., Monteith P., Noble N.A., Unser A.B., Daita K., Fisher A.R. (2014). Altered profile of human gut microbiome is associated with cirrhosis and its complications. J. Hepatol..

[130] Tripathi A., Debelius J., Brenner D.A., Karin M., Loomba R., Schnabl B., Knight R. (2018). The gut-liver axis and the intersection with the microbiome. Nat. Rev. Gastroenterol. Hepatol..

[131] Bajaj J.S., Jakab S.S., Jesudian A.B., Rahimi R.S., Duarte-Rojo A., Chen P.-H., Wong R.J., Tapper E.B., Tandon P. (2026). ACG Clinical Guideline: Hepatic Encephalopathy. Am. J. Gastroenterol..

[132] Thévenot T., Elkrief L., Bureau C., Bardou-Jacquet E., Rosa I., Nguyen-Khac E., Oberti F., Pitta A., Mallet M., Lebossé F. (2025). Effect of rifaximin in patients with severe cirrhosis and ascites: A randomized double-blind placebo-controlled trial. J. Hepatol..

[133] Caraceni P., Vargas V., Solà E., Alessandria C., de Wit K., Trebicka J., Angeli P., Mookerjee R.P., Durand F., Pose E. (2021). The Use of Rifaximin in Patients With Cirrhosis. Hepatology.

[134] Huang L., Yu Q., Peng H., Zhen Z. (2022). Alterations of gut microbiome and effects of probiotic therapy in patients with liver cirrhosis: A systematic review and meta-analysis. Medicine.

[135] Bajaj J.S., Fagan A., Gavis E.A., Sterling R.K., Gallagher M.L., Lee H., Matherly S.C., Siddiqui M.S., Bartels A., Mousel T. (2025). Microbiota transplant for hepatic encephalopathy in cirrhosis: The THEMATIC trial. J. Hepatol..

[136] Lombardi M., Troisi J., Motta B.M., Torre P., Masarone M., Persico M. (2024). Gut-Liver Axis Dysregulation in Portal Hypertension: Emerging Frontiers. Nutrients.

[137] Anand S., Mande S.S. (2022). Host-microbiome interactions: Gut-Liver axis and its connection with other organs. NPJ Biofilms Microbiomes.

[138] Garcia-Tsao G., Abraldes J.G., Rich N.E., Wong V.W.-S. (2024). AGA Clinical Practice Update on the Use of Vasoactive Drugs and Intravenous Albumin in Cirrhosis: Expert Review. Gastroenterology.

[139] Silvey S., Patel N., Tsai S.Y., Nadeem M., Sterling R.K., Markley J.D., French E., O’Leary J.G., Bajaj J.S. (2025). Higher Rate of Spontaneous Bacterial Peritonitis Recurrence With Secondary Spontaneous Bacterial Peritonitis Prophylaxis Compared With No Prophylaxis in 2 National Cirrhosis Cohorts. Am. J. Gastroenterol..

[140] Lontos S., Gow P.J., Vaughan R.B., Angus P.W. (2008). Norfloxacin and trimethoprim-sulfamethoxazole therapy have similar efficacy in prevention of spontaneous bacterial peritonitis. J. Gastroenterol. Hepatol..

[141] Alvarez R.F., de Mattos A.A., Corrêa E.B.D., Cotrim H.P., Nascimento T.V.S.B. (2005). Trimethoprim-sulfamethoxazole versus norfloxacin in the prophylaxis of spontaneous bacterial peritonitis in cirrhosis. Arq. Gastroenterol..

[142] Iyer U., Jara-Tantoco M.N., Delgado A. (2026). P-163. Trimethoprim-Sulfamethoxazole is Associated with Decreased Mortality Compared to Fluoroquinolones for Secondary Prophylaxis of Spontaneous Bacterial Peritonitis: A Global Network Analysis. Open Forum Infect. Dis..

[143] Bajaj J.S., Rodriguez M.P., Fagan A., McGeorge S., Sterling R.K., Lee H., Luketic V., Fuchs M., Davis B.C., Sikaroodi M. (2022). Impact of bacterial infections and spontaneous bacterial peritonitis prophylaxis on phage-bacterial dynamics in cirrhosis. Hepatology.

[144] Juncu S., Minea H., Lungu A., Jucan A., Avram R., Buzuleac A.-M., Cojocariu C., Diaconu L.S., Stanciu C., Trifan A. (2025). Fluoroquinolones for the Prophylaxis of Spontaneous Bacterial Peritonitis in Patients with Liver Cirrhosis: Are They Losing Ground?. Life.

[145] Salehi S., Tranah T.H., Lim S., Heaton N., Heneghan M., Aluvihare V., Patel V.C., Shawcross D.L. (2019). Rifaximin reduces the incidence of spontaneous bacterial peritonitis, variceal bleeding and all-cause admissions in patients on the liver transplant waiting list. Aliment. Pharmacol. Ther..

[146] Fernández J., Prado V., Trebicka J., Amoros A., Gustot T., Wiest R., Deulofeu C., Garcia E., Acevedo J., Fuhrmann V. (2019). Multidrug-resistant bacterial infections in patients with decompensated cirrhosis and with acute-on-chronic liver failure in Europe. J. Hepatol..

[147] Liu J., Guevara J.G., Macnaughtan J., Jin Y., Clasen F., Kerbert A., Portlock T., González J.M., Habtesion A., Phillips A. (2025). TOP-218 Yaq-001 positively impacts gut microbiome composition, virulence, antimicrobial resistance gene profile resulting in significant effects on ammonia, endotoxemia and inflammation in cirrhosis patients. J. Hepatol..

[148] Liu J., Macnaughtan J., Kerbert A.J.C., Portlock T., Martínez Gonzalez J., Jin Y., Clasen F., Habtesion A., Ji H., Jin Q. (2024). Clinical, experimental and pathophysiological effects of Yaq-001: A non-absorbable, gut-restricted adsorbent in models and patients with cirrhosis. Gut.

[149] Bajaj J.S., Fagan A., Gavis E.A., Kassam Z., Sikaroodi M., Gillevet P.M. (2019). Long-term Outcomes of Fecal Microbiota Transplantation in Patients With Cirrhosis. Gastroenterology.

[150] University of Chicago The Microbiota Augmentation to Reestablish Commensal Organisms (MARCO) Trial. https://clinicaltrials.gov/study/NCT06871111.

[151] Brödel A.K., Charpenay L.H., Galtier M., Fuche F.J., Terrasse R., Poquet C., Havránek J., Pignotti S., Krawczyk A., Arraou M. (2024). In situ targeted base editing of bacteria in the mouse gut. Nature.

[152] Lam K.N., Spanogiannopoulos P., Soto-Perez P., Alexander M., Nalley M.J., Bisanz J.E., Nayak R.R., Weakley A.M., Yu F.B., Turnbaugh P.J. (2021). Phage-delivered CRISPR-Cas9 for strain-specific depletion and genomic deletions in the gut microbiome. Cell Rep..

[153] Nath A., Bhattacharjee R., Nandi A., Sinha A., Kar S., Manoharan N., Mitra S., Mojumdar A., Panda P.K., Patro S. (2022). Phage delivered CRISPR-Cas system to combat multidrug-resistant pathogens in gut microbiome. Biomed. Pharmacother..

[154] Duan Y., Llorente C., Lang S., Brandl K., Chu H., Jiang L., White R.C., Clarke T.H., Nguyen K., Torralba M. (2019). Bacteriophage targeting of gut bacterium attenuates alcoholic liver disease. Nature.

[155] Bloom P.P., Tapper E.B., Young V.B., Lok A.S. (2021). Microbiome therapeutics for hepatic encephalopathy. J. Hepatol..

[156] Zhou Y.-L., Pu S.-T., Xiao J.-B., Luo J., Xue L. (2024). Meta-analysis of probiotics efficacy in the treatment of minimum hepatic encephalopathy. Liver Int..

[157] Dalal R., McGee R.G., Riordan S.M., Webster A.C. (2017). Probiotics for people with hepatic encephalopathy. Cochrane Database Syst. Rev..

[158] Rose E.C., Odle J., Blikslager A.T., Ziegler A.L. (2021). Probiotics, Prebiotics and Epithelial Tight Junctions: A Promising Approach to Modulate Intestinal Barrier Function. Int. J. Mol. Sci..

[159] Montagnese S., Rautou P.E., Romero-Gómez M., Larsen F.S., Shawcross D.L., Thabut D., Vilstrup H., Weissenborn K. (2022). EASL Clinical Practice Guidelines on the management of hepatic encephalopathy. J. Hepatol..

[160] Úbeda M., Lario M., Muñoz L., Borrero M.-J., Rodríguez-Serrano M., Sánchez-Díaz A.-M., Del Campo R., Lledó L., Pastor Ó., García-Bermejo L. (2016). Obeticholic acid reduces bacterial translocation and inhibits intestinal inflammation in cirrhotic rats. J. Hepatol..

[161] Trebicka J., Macnaughtan J., Schnabl B., Shawcross D.L., Bajaj J.S. (2021). The microbiota in cirrhosis and its role in hepatic decompensation. J. Hepatol..

[162] Fiorucci S., Urbani G., Distrutti E., Biagioli M. (2025). Obeticholic Acid and Other Farnesoid-X-Receptor (FXR) Agonists in the Treatment of Liver Disorders. Pharmaceuticals.

[163] Ma K., Tang D., Yu C., Zhao L. (2021). Progress in research on the roles of TGR5 receptor in liver diseases. Scand. J. Gastroenterol..

[164] Canovai E., Farré R., Accarie A., Lauriola M., de Hertogh G., Vanuytsel T., Pirenne J., Ceulemans L.J. (2023). INT-767-A Dual Farnesoid-X Receptor (FXR) and Takeda G Protein-Coupled Receptor-5 (TGR5) Agonist Improves Survival in Rats and Attenuates Intestinal Ischemia Reperfusion Injury. Int. J. Mol. Sci..

[165] Rizzo G., Passeri D., de Franco F., Ciaccioli G., Donadio L., Rizzo G., Orlandi S., Sadeghpour B., Wang X.X., Jiang T. (2010). Functional characterization of the semisynthetic bile acid derivative INT-767, a dual farnesoid X receptor and TGR5 agonist. Mol. Pharmacol..

[166] Liu W., Hu D., Huo H., Zhang W., Adiliaghdam F., Morrison S., Ramirez J.M., Gul S.S., Hamarneh S.R., Hodin R.A. (2016). Intestinal Alkaline Phosphatase Regulates Tight Junction Protein Levels. J. Am. Coll. Surg..

[167] Liu Y., Cavallaro P.M., Kim B.-M., Liu T., Wang H., Kühn F., Adiliaghdam F., Liu E., Vasan R., Samarbafzadeh E. (2021). A role for intestinal alkaline phosphatase in preventing liver fibrosis. Theranostics.

[168] Liu Y., Lv H., Zhang H., Li H., Wang A., Yu S., He Q., Chen S., Wang J., Ran X. (2026). The protective role of intestinal alkaline phosphatase in inflammatory bowel disease-associated non-alcoholic fatty liver disease. Life Sci..

[169] Fogacci F., Giovannini M., Di Micoli V., Grandi E., Borghi C., Cicero A.F.G. (2024). Effect of Supplementation of a Butyrate-Based Formula in Individuals with Liver Steatosis and Metabolic Syndrome: A Randomized Double-Blind Placebo-Controlled Clinical Trial. Nutrients.

[170] Abbasi F., Haghighat Lari M.M., Khosravi G.R., Mansouri E., Payandeh N., Milajerdi A. (2024). A systematic review and meta-analysis of clinical trials on the effects of glutamine supplementation on gut permeability in adults. Amino Acids.

[171] Achamrah N., Déchelotte P., Coëffier M. (2017). Glutamine and the regulation of intestinal permeability: From bench to bedside. Curr. Opin. Clin. Nutr. Metab. Care.

[172] Cordes F., Demmig C., Bokemeyer A., Brückner M., Lenze F., Lenz P., Nowacki T., Tepasse P., Schmidt H.H., Schmidt M.A. (2020). MicroRNA-320a Monitors Intestinal Disease Activity in Patients With Inflammatory Bowel Disease. Clin. Transl. Gastroenterol..

[173] Tili E., Michaille J.-J., Piurowski V., Rigot B., Croce C.M. (2017). MicroRNAs in intestinal barrier function, inflammatory bowel disease and related cancers-their effects and therapeutic potentials. Curr. Opin. Pharmacol..

